# SRSF1 suppresses selection of intron-distal 5′ splice site of *DOK7* intron 4 to generate functional full-length Dok-7 protein

**DOI:** 10.1038/s41598-017-11036-z

**Published:** 2017-09-05

**Authors:** Khalid Bin Ahsan, Akio Masuda, Mohammad Alinoor Rahman, Jun-ichi Takeda, Mohammad Nazim, Bisei Ohkawara, Mikako Ito, Kinji Ohno

**Affiliations:** Division of Neurogenetics, Center for Neurological Diseases and Cancer, Nagoya University Graduate School of Medicine, Nagoya, Aichi Japan

## Abstract

Dok-7 is a non-catalytic adaptor protein that facilitates agrin-induced clustering of acetylcholine receptors (AChR) at the neuromuscular junction. Alternative selection of 5′ splice sites (SSs) of *DOK7* intron 4 generates canonical and frame-shifted transcripts. We found that the canonical full-length Dok-7 enhanced AChR clustering, whereas the truncated Dok-7 did not. We identified a splicing *cis*-element close to the 3′ end of exon 4 by block-scanning mutagenesis. RNA affinity purification and mass spectrometry revealed that SRSF1 binds to the *cis*-element. Knocking down of *SRSF1* enhanced selection of the intron-distal 5′ SS of *DOK7* intron 4, whereas MS2-mediated artificial tethering of SRSF1 to the identified *cis*-element suppressed it. Isolation of an early spliceosomal complex revealed that SRSF1 inhibited association of U1 snRNP to the intron-distal 5′ SS, and rather enhanced association of U1 snRNP to the intron-proximal 5′ SS, which led to upregulation of the canonical *DOK7* transcript. Integrated global analysis of CLIP-seq and RNA-seq also indicated that binding of SRSF1 immediately upstream to two competing 5′ SSs suppresses selection of the intron-distal 5′ SS in hundreds of human genes. We demonstrate that SRSF1 critically regulates alternative selection of adjacently placed 5′ SSs by modulating binding of U1 snRNP.

## Introduction

Alternative splicing is a ubiquitous mechanism to achieve transcriptome and proteome diversity. Recent studies based on high-throughput sequencing revealed that around 95% of the human genes are alternatively spliced to produce various transcript isoforms^1^. Inaccurate regulation of splicing often causes generation of aberrant mRNA that may code for a defective protein with compromised function^2, 3^, and may cause degradation of the mRNA through non-sense mediated mRNA decay (NMD) pathway^4^. The splicing process is carried out by the spliceosome, which is the large and complex molecular machinery comprised of small nuclear RNAs (snRNAs) and proteins^5, 6^. In higher eukaryotes, introns are defined by three essential but degenerative elements: the 5′ splice site (5′ SS), the branch point, and the 3′ splice site. Recognition of these elements by small nuclear ribonucleoprotiens (snRNPs) is the first step for efficient formation of an early spliceosomal complex and for correct splicing^7, 8^.

Splice site-like elements are frequently observed in human genome^9^. The selection of a particular splice site among several options is dependent on various factors, including the strength of splice sites^10^, architecture of pre-mRNAs^11^, intrinsic *cis*-acting elements^12^, cognate *trans*-acting factors^12^, RNA secondary structure^13^, and transcription rate of RNA polymerase II^14^. Previous studies showed that RNA-binding proteins, SRSF1 and hnRNP A1, activate the selection of intron-proximal 5′ SS^15^ and intron-distal 5′ SS^16^, respectively. Similarly, an exonic splicing enhancer (ESE) and an exonic splicing silencer (ESS) located between two competing 5′ SSs facilitate and suppress selection of the intron-proximal 5′ SS, respectively. Indeed, tethering of SRSF1 between the competing 5′ SSs facilitates selection of the intron-proximal 5′ SS^17^. Additionally, SRSF2^18^, SRSF5^18^, hnRNP A1^17, 19^ and TIA-1^12^ bind to and activate ESE or ESS around the 5′ SSs. SRSF1 is a member of serine and arginine rich (SR) protein family, which regulates multiple steps of RNA processing, including pre-mRNA splicing^20^, transcription, mRNA stability, nuclear export, NMD, and protein translation^21^.

**Figure 1:**
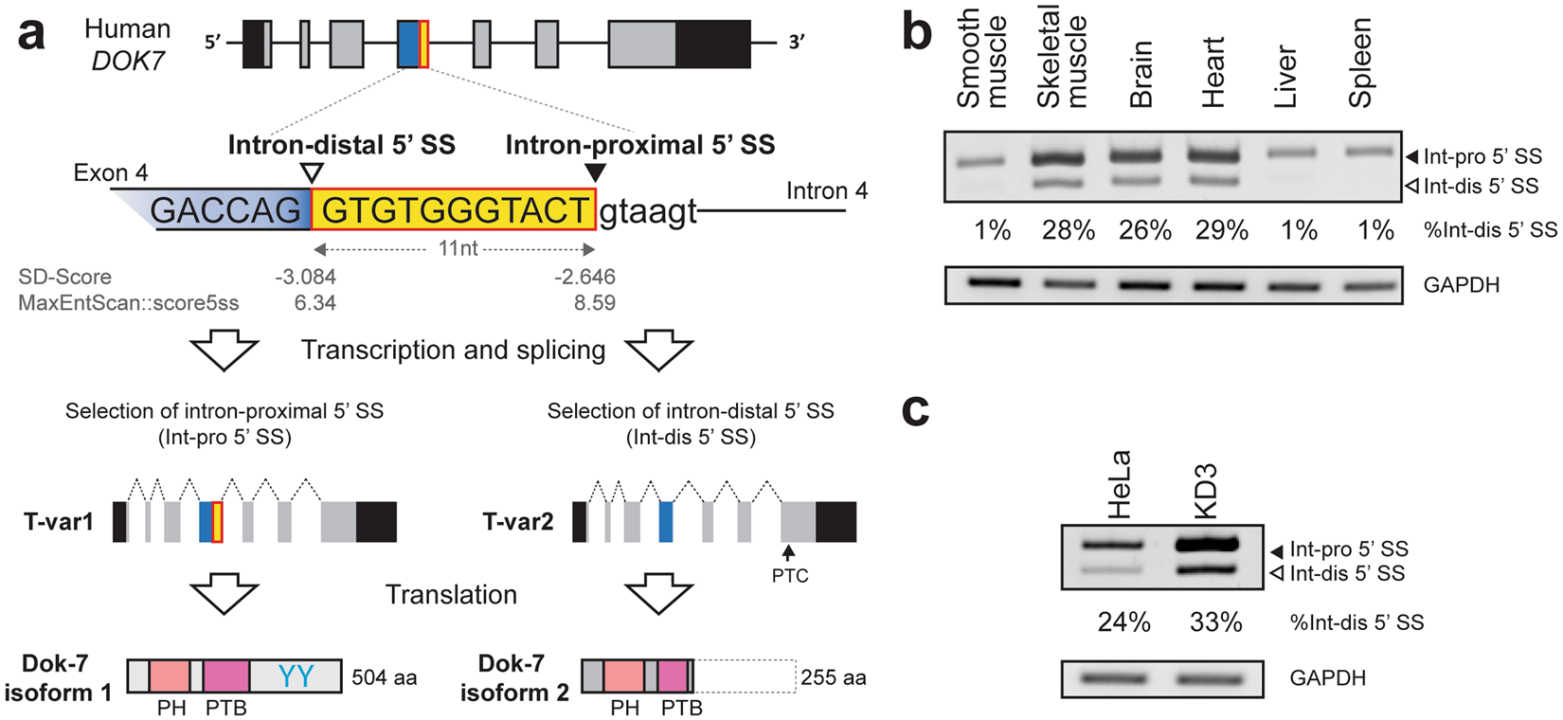
Schematic of alternative splicing of *DOK7*, and its splicing pattern in human tissues and cell lines. (**a**) Schematic of genomic structure of human *DOK7* gene locus. Exons and introns are shown in boxes and solid lines, respectively. Human *DOK7* intron 4 contains two 5′ SSs, which are 11 nucleotides apart. Open and closed arrowheads point to the intron-distal and intron-proximal 5′ SSs, respectively. Constitutive segment of exon 4 is indicated by a blue box, and alternatively spliced segment of exon 4 is indicated by a yellow box. Transcript variant 1 (T-var1) and transcript variant 2 (T-var2) are schematically shown below the gene structure. Position of the premature terminal codon (PTC) in T-var2 is indicated by an arrow. T-var1 encodes the canonical full-length Dok-7 isoform 1 containing the PH and PTB domains, as well as two tyrosine residues (YY) that are target motifs of SH2 domain. T-var2 encodes the truncated Dok-7 isoform 2 retaining the PH domain and part of the PTB domain, but lacking two tyrosine residues. (**b**) RT-PCR showing alternative 5′ SS selection of *DOK7* intron 4 in human tissues. *DOK7* transcripts are highly expressed in the skeletal muscle, brain, and heart, in which both the intron-proximal 5′ SS (Int-pro 5′ SS) and the intron-distal 5′ SS (Int-dis 5′ SS) are selected. (**c**) RT-PCR showing alternative 5′ SS selection of *DOK7* intron 4 in HeLa cells and immortalized human myogenic KD3 cells.

Dok-7, a member of Dok protein family is a non-catalytic cytoplasmic adapter protein. It is abundantly expressed in the skeletal muscle and is essential for formation of the neuromuscular junction^22^. Knocking out of *Dok7* compromises formation of the neuromuscular junction (NMJ) in mice^23^, and mutations in *DOK7* cause congenital myasthenic syndrome (CMS) in humans^24, 25^. Although the regulatory mechanisms of alternative splicing of *DOK7* have not been dissected in detail, annotation databases of the human genome including RefSeq, Ensemble, and AceView indicate that intron 4 of the human *DOK7* gene harbors two 5′ SSs (Fig. 1a), which are 11 nucleotides apart. Selection of the intron-proximal 5′ SS generates a long transcript variant 1 (T-var1) encoding the canonical Dok-7 protein (Dok-7 isoform 1). In contrast, selection of the intron-distal 5′ SS generates a frame-shifted transcript variant 2 (T-var2) encoding a truncated Dok-7 protein (Dok-7 isoform 2) lacking two tyrosine residues, which are target motifs of the Src homology 2 (SH2) domain and are indispensable for clustering of acetylcholine receptors (AChR) at the neuromuscular junction^26^ (Fig. 1a).

In the present study, we dissected the underlying mechanisms that regulate selection of the two competing 5′ SSs at *DOK7* intron 4. We identified that a *trans*-acting factor, SRSF1, suppresses selection of the intron-distal 5′ SS through binding to the splicing regulatory *cis*-element located closely upstream of these 5′ SSs. Further mechanistic analysis demonstrated that SRSF1 inhibits the assembly of U1 snRNP on the intron-distal 5′ SS leading to the generation of *DOK7* T-var1.

## Results

### *DOK7* intron 4 is alternatively spliced at 5′ SS

Since annotation databases indicate existence of two 5′ SSs in human *DOK7* intron 4, we initially examined differential selections of these two sites by RT-PCR of total RNA extracted from various human tissues and cell lines (Fig. 1b and c). We observed abundant expressions of *DOK7* transcripts in the skeletal muscle, brain and, heart (Fig. 1b) in agreement with a previous report^23^, and found that the intron-distal 5′ SS was selected in nearly a quarter of *DOK7* transcripts in these three tissues. Similar alternative selection of the intron-distal 5′ SS of *DOK7* intron 4 was observed in immortalized human myogenic KD3 cells and HeLa cells (Fig. 1c). In contrast, *DOK7* intron 4 was constitutively spliced in the smooth muscle, liver, and spleen (Fig. 1b).

### Selection of the intron-distal 5′ SS of *DOK7* intron 4 generates a non-functional Dok-7 lacking AChR clustering activity

**Figure 2:**
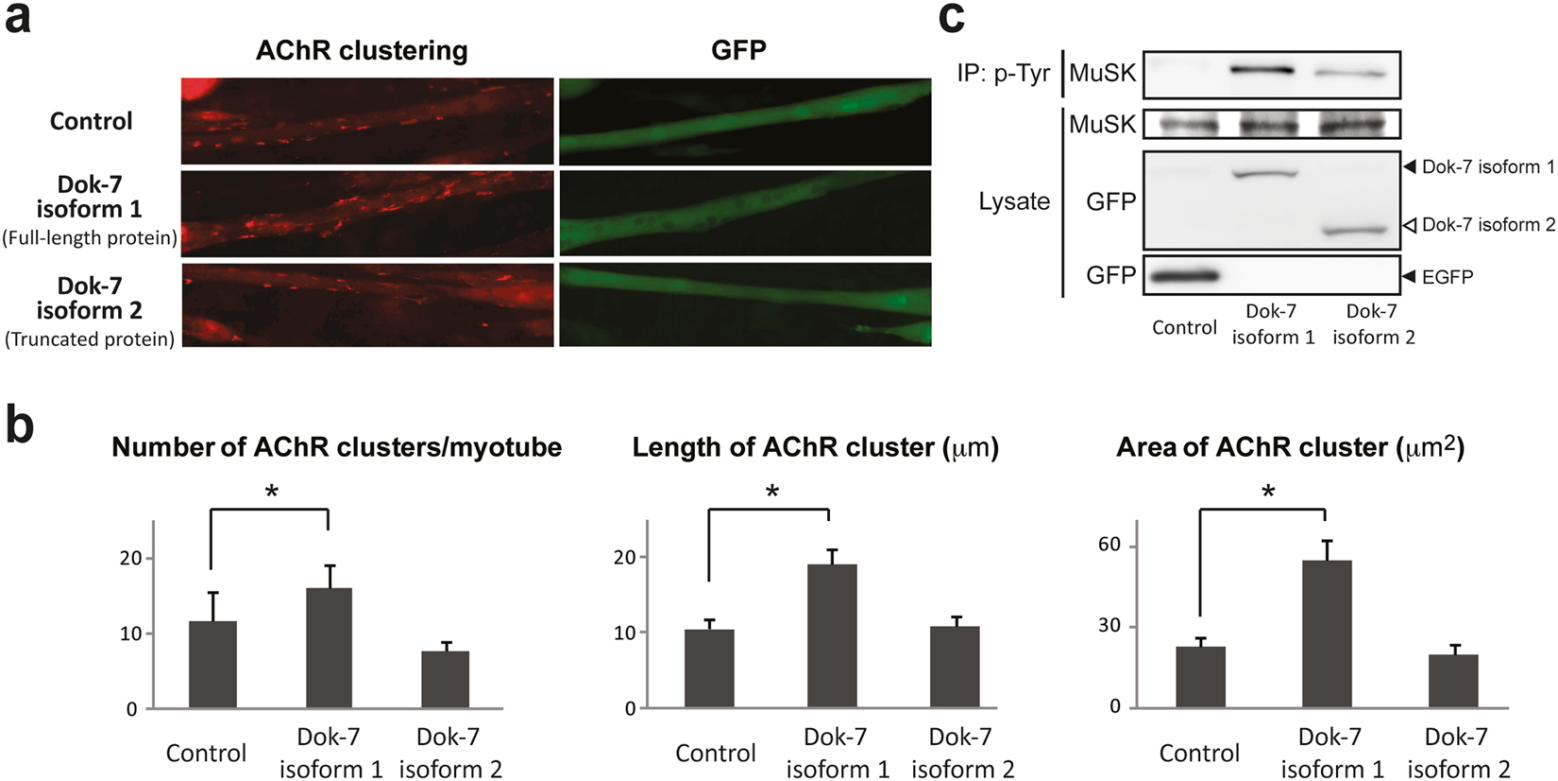
Functional assay of Dok-7 isoforms 1 and 2 in AChR clustering. (**a**) Enhancement of agrin-mediated AChR clustering of C2C12 myotubes by overexpression of Dok-7 isoforms 1 and 2. C2C12 myoblasts are transfected with cDNA encoding Dok-7 isoform 1 or 2 fused with GFP, or cDNA encoding GFP alone (Control), and are cultured in differentiation medium. On day 5, 10 ng/ml agrin is added to induce AChR clustering, and AChR clustering is visualized with Alexa594-conjugated α-bungarotoxin at 12 h later. Transfected C2C12 myotubes are identified by GFP. (**b**) Morphometric analysis showing that Dok-7 isoform 1, but not isoform 2, induces AChR clustering. The mean and S.E. are indicated. *p* < 0.05 (not indicated) by one-way ANOVA for all datasets. *p < 0.05 by post-hoc Tukey HSD test. (**c**) MuSK phosphorylation assay of differentiation-induced C2C12 myoblasts transfected with cDNA encoding Dok-7 isoform 1 or 2 fused with GFP, or cDNA encoding GFP alone (Control), along with cDNA encoding FLAG-tagged MuSK. Phosphorylated MuSK is detected by immunoprecipitation of cell lysate by anti-phosphotyrosine antibody (p-Tyr) followed by immunoblotting with anti-FLAG antibody (MuSK).

Selection of the intron-proximal 5′ SS generates the canonical Dok-7 protein (isoform 1), which is essential for enhancing clustering of AChRs, whereas selection of the intron-distal 5′ SS causes a frame-shift in the middle of *DOK7* and generates a truncated Dok-7 protein (isoform 2), which partially disrupts the PTB domain and lacks two tyrosine residues targeted by the SH2 domain (Fig. 1a). To examine whether isoform 2 retains a biological function to enhance AChR clustering, we made two constructs expressing isoform 1 and isoform 2 fused with EGFP, and overexpressed each of them in C2C12 myoblasts. After myotube differentiation of C2C12 cells, agrin was added to the culture medium, and cells were stained with α-bungarotoxin to observe AChR clusters. We found that overexpression of isoform 1 significantly enhanced AChR clustering, as previously reported^22^, while isoform 2 has a no effect on AChR clustering (Fig. 2a). Consistently, overexpression of isoform 1, but not of isoform 2, increased the number, length, and area of AChR clusters (Fig. 2b). Phosphorylation of MuSK was markedly induced by overexpression of isoform 1, as previously reported^23^, whereas induction of MuSK phosphorylation was insufficient with isoform 2 (Fig. 2c). These results suggest that selection of the intron-distal 5′ SS results in production of a non-functional Dok-7 retaining minimal MuSK phosphorylation activity with diminished AChR clustering activity.

### Construction of minigenes to characterize two competitive 5′ SSs

**Figure 3:**
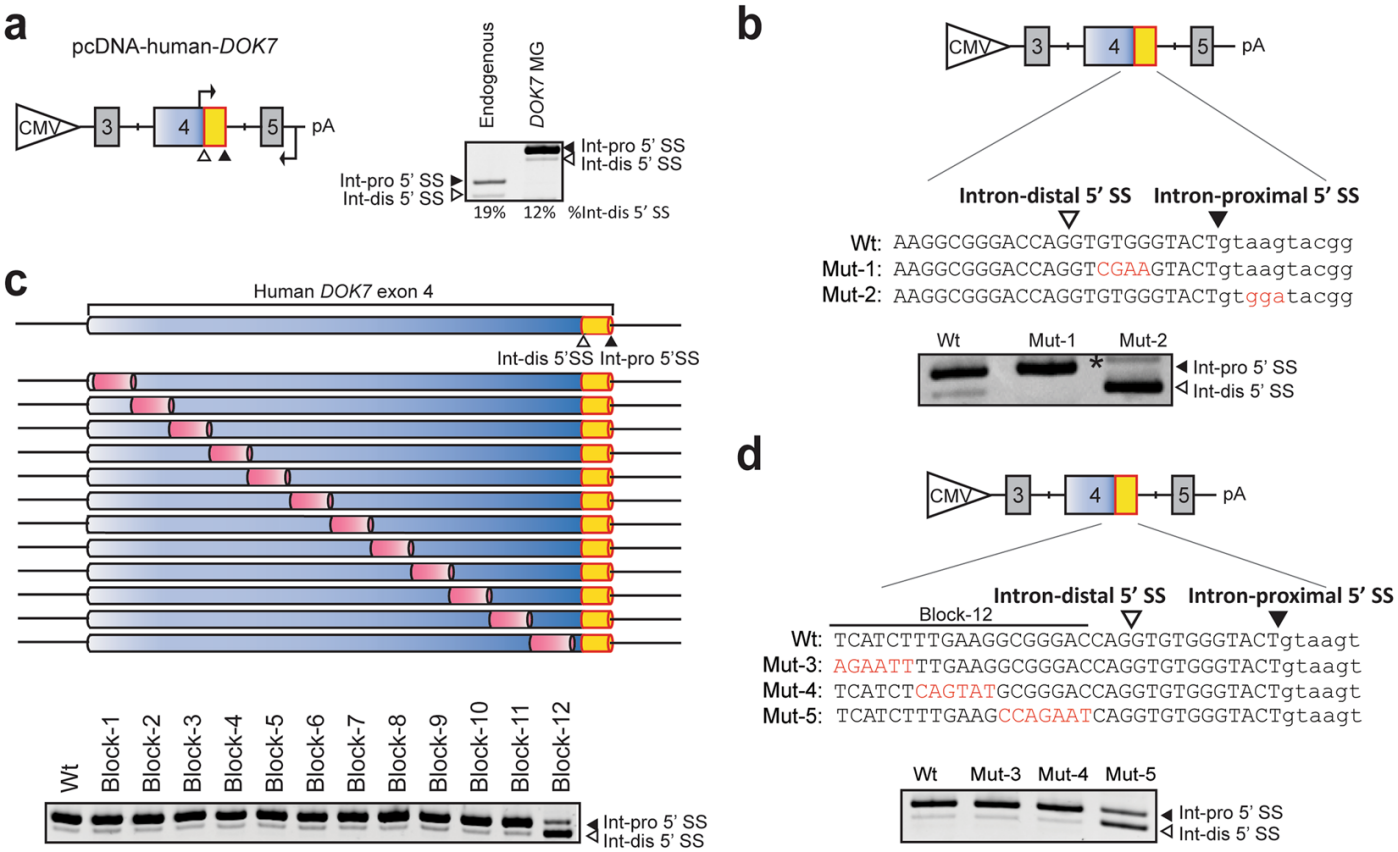
Identification of a splicing regulatory *cis*-element in *DOK7* exon 4. (**a**) Structure of human *DOK7* minigene spanning exons 3 to 5 driven by a CMV promoter (pcDNA-human-*DOK7*). Positions of intron-distal (Int-dis) and intron-proximal (Int-pro) 5′ SS are indicated by open and closed arrowheads, respectively. Positions of primers for RT-PCR are indicated by bent arrows. RT-PCR of endogenous *DOK7* transcripts and *DOK7* minigene transcripts (*DOK7* MG) in HeLa cells are shown in the right panel. Percentages of the selection of intron-distal 5′ SS are shown at the bottom. (**b**) Schematic of pcDNA-human-*DOK7* minigenes carrying wild-type (Wt) and mutant (Mut-1 and -2) 5′ SSs. Introduced mutations are shown in red. RT-PCR of wild-type and mutant constructs transfected in HeLa cells are shown. A transcript generated by activation of a cryptic splice site at IVS4 + 12 (not shown) in intron 4 is indicated by an asterisk. (**c**) Schematic of *cis*-regulatory block-scanning mutagenesis of *DOK7* exon 4 in the context of pcDNA-human-*DOK7* minigene. A 15-nucleotide heterologous sequence of 5′-TCAGTATGACTCTCA-3′ is introduced into each block, excluding Block-12, which is replaced with 19-nucleotide heterologous sequence of 5′-TCAGTATGACTCTCAGTAT-3′. No mutations are introduced into the first three nucleotides and the last fourteen nucleotides of exon 4, where the intron-distal 5′ SS is located. Positions of intron-distal (Int-dis) and intron-proximal (Int-pro) 5′ SS are indicated by open and closed arrowheads, respectively. RT-PCR of these constructs in HeLa cells are shown below. (**d**) Schematic of pcDNA-human-*DOK7* minigenes carrying wild-type (Wt) and mutant (Mut-3, -4, and -5) sequence. RT-PCR of wild-type and mutant constructs transfected in HeLa cells are shown. (**b**,**d**) Mutant nucleotides are indicated in red. Uppercase and lowercase letters represent exonic and intronic nucleotides, respectively.

To dissect the underlying mechanisms of alternative 5′ SS selection of *DOK7* intron 4, we constructed a human *DOK7* minigene spanning exons 3 to 5 in pcDNA3.1+ mammalian expression vector (Fig. 3a). As expected, RT-PCR analysis of HeLa cells transfected with this minigene demonstrated a similar splicing pattern to that we observed with endogenous transcripts (Fig. 3a, right panel).

Several algorithms efficiently predict strength of 5′ SS^10, 27^. The strength of the intron-proximal 5′ SS (SD score −2.646 and MaxEntScan::score5ss 8.59) is higher than that of the intron-distal 5′ SS (SD score −3.084, MaxEntScan::score5ss 6.34). We made two mutant constructs, Mut-1 and Mut-2, in which splicing strengths of the intron-distal and intron-proximal 5′ SS, respectively, were drastically weakened, while a GT dinucleotide was retained. SD-scores of the mutated 5′ SS in Mut-1 and Mut-2 were reduced to −5.277 and −4.976, respectively. Similarly, MaxEntScan::score5ss of the mutated 5′ SS in Mut-1 and Mut-2 were reduced to −0.30 and −9.13, respectively. RT-PCR analysis of HeLa cells transfected with these constructs showed that the intact 5′ SSs were exclusively selected in both constructs (Fig. 3b). These results suggest that the two 5′ SSs in *DOK7* intron 4 are competing for splicing each other, although the intron-proximal 5′ SS is preferentially selected.

### Identification of a splicing regulatory *cis*-element that modulates alternative selection of 5′ SS of *DOK7* intron 4

Splicing *cis-*elements around 5′ SSs play a crucial role in the selection of alternative 5′ SSs^28^. To identify splicing regulatory *cis*-element(s) that regulate the selection of 5′ SS of *DOK7* intron 4, we scanned the entire exon 4 by substituting 12 blocks excluding the first three nucleotides and the last fourteen nucleotides, which contained the intron-distal 5′ SS. We sequentially introduced a 15-nucleotide heterologous sequence block (5′-TCAGTATGACTCTCA-3′) into the first 11 blocks (Blocks-1 to -11) and a 19-nucleotide heterologous sequence block (5′-TCAGTATGACTCTCAGTAT-3′) for Block-12 of the minigene (Fig. 3c). The introduced sequence blocks were previously reported to have no effect on splicing^29, 30^. These constructs were transfected into HeLa cells, and selection of 5′ SSs was examined by RT-PCR. Disruption of Block-12 prominently enhanced selection of the intron-distal 5′ SS (Fig. 3c, lower panel). We also introduced the 15-nucleotide splicing-neutral sequence block into the last 2 blocks (Blocks-13 and -14 in Supplementary Fig. S1). We found that disruption of Block-14, but not Block-13, enhanced selection of the intron-distal 5′ SS (Supplementary Fig. S1). These results suggest presence of a splicing regulatory *cis*-element in Block-12. To characterize the minimal essential sequences in Block-12, we further mutated a segment of six or seven nucleotides in Block-12 (Mut-3, -4, and -5 in Fig. 3d). We confirmed that the mutated segments did not *de novo* gain binding of an RNA-binding protein according to SpliceAid2, which is a web service program to predict the binding sites of RNA-binding proteins for a given RNA sequence based on a database derived from experimentally proven RNA-binding protein-recognition sites^31^. We found that substitution of the 3′ third of Block-12 (Mut-5), but not the 5′ or the middle thirds (Mut-3 or Mut-4), altered the 5′ SS selection (Fig. 3d). Thus, the Mut-5 region in Block-12 close to the 3′ end of exon 4 harbors a critical splicing regulatory *cis*-element.

### SRSF1 binds to the identified splicing regulatory *cis*-element

**Figure 4:**
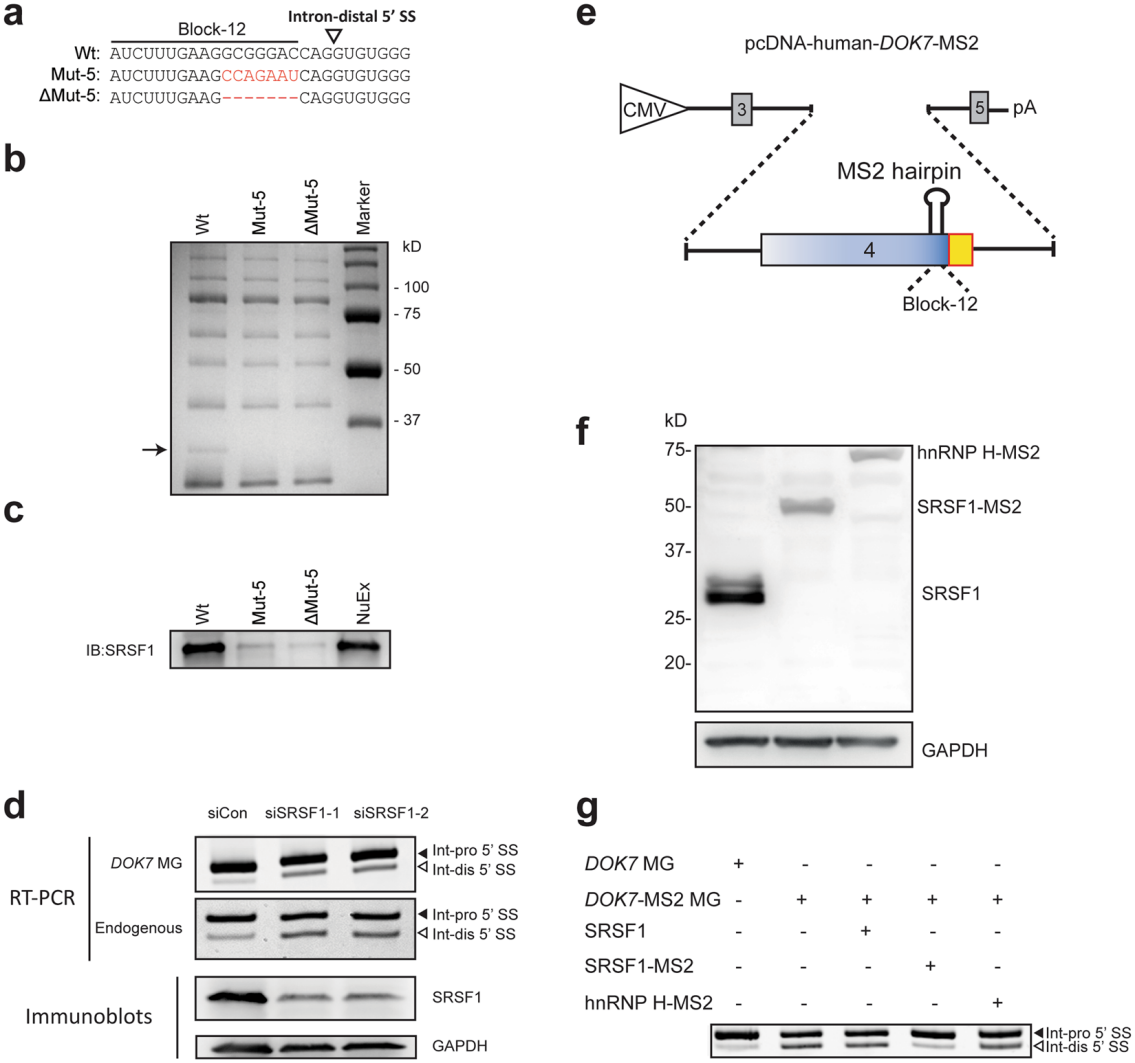
SRSF1 binds to the identified *cis*-element and facilitates selection of the intron-proximal 5′ SS of *DOK7* intron 4. (**a**) RNA probes carrying wild-type (Wt), mutant (Mut-5), and partially deleted (ΔMut-5) sequences. (**b**) Coomassie blue staining of RNA affinity-purified products using HeLa nuclear extract with the indicated biotinylated RNA probes. A single protein band at ~30 kDa (black arrow) is associated with Wt probe, but not with Mut-5 or ΔMut-5 probe. Mass spectrometry analysis revealed that the identity of this protein is SRSF1. The other bands commonly observed in the Wt, Mut-5 and ΔMut-5 RNA probes are repeatedly identified in our RNA affinity purification analyses^43, 45, 55^. Mass spectrometry analysis of these bands revealed that none of the identified proteins carry an RNA-recognition motif, suggesting that these bands are likely due to non-specific binding of proteins to the streptavidin-sepharose beads. NuEx, HeLa nuclear extract. (**c**) Immunoblotting of RNA affinity-purified proteins in panel (b) with anti-SRSF1 antibody. (**d**) Upper panels show RT-PCR of *DOK7* minigene (*DOK7* MG) encoded by pcDNA-human-*DOK7* and endogenous *DOK7* transcripts in HeLa cells treated with two different siRNAs against *SRSF1* (siSRSF1-1 and siSRSF1-2). Lower panels show immunoblotting with indicated antibodies after *SRSF1*-knockdown with two different siRNAs in HeLa cells. (**e**) Schematic of pcDNA-human-*DOK7*-MS2 minigene. Block-12 sequence is substituted with the MS2 coat protein-binding hairpin RNA sequence. (**f**) Immunoblotting with anti-His antibody to show overexpression of His-tagged transgene products in HeLa cells: SRSF1, SRSF1 fused with MS2 coat protein (SRSF1-MS2), and hnRNP H fused with MS2 coat protein (hnRNP H-MS2). (**g**) RT-PCR of *DOK7* minigene (*DOK7* MG) and *DOK7*-MS2 minigene (*DOK7*-MS2 MG) encoded by pcDNA-human-*DOK7* and pcDNA-human-*DOK7-*MS2, respectively, in HeLa cells co-transfected with the indicated effectors. Int-pro 5′ SS and Int-dis 5′ SS point to transcripts generated by selection of the intron-proximal and intron-distal 5′ SSs, respectively.

A lot of splicing factors including hnRNP and SR proteins regulate selection of alternative 5′ SS through recognition of nearby *cis*-elements^12, 18, 19^. To identify a *trans*-acting factor that binds to the identified *cis*-element, we performed an RNA affinity purification assay using biotinylated RNA probe including the wild-type Block-12 and HeLa nuclear extract (Fig. 4a). Our analysis identified one distinct band of ~30 kDa that was associated with an RNA probe for the wild-type Block-12 (Wt), but not for the nucleotide-substituted mutant (Mut-5) or the deletion mutant (ΔMut-5) (Fig. 4b). Mass spectrometry analysis of the excised band disclosed that the identified band was SRSF1, which was also confirmed by immunoblotting using antibody against SRSF1 (Fig. 4c). Indeed, the highest ESEfinder score^32, 33^ of SRSF1 was 1.74 at GGGACCA in the Wt probe, whereas it was reduced to −3.77 at AGAATCA in the Mut-5 probe.

Real-time RT-PCR of *SRSF1* mRNA in various human tissues revealed that *SRSF1* mRNA is abundantly expressed in smooth muscle, liver and spleen (Supplementary Fig. S2), where the selection of intron-distal 5′ splice site of *DOK7* intron 4 is suppressed (Fig. 1b). In contrast, the expression levels of *SRSF1* mRNA are low in skeletal muscle, brain and heart (Supplementary Fig. S2), where the selection of intron-distal 5′splice site of *DOK7* intron 4 is facilitated (Fig. 1b), suggesting that SRSF1 expression is inversely correlated with the selection of intron-distal 5′ SSs of *DOK7* intron 4.

### Binding of SRSF1 to the *cis*-element activates the intron-distal 5′ SS and suppresses the intron-proximal 5′ SS of *DOK7* intron 4

We next asked whether binding of SRSF1 to the identified *cis*-element indeed regulates alternative 5′ SS selection of *DOK7* intron 4 *in cellulo*. We knocked down endogenous *SRSF1* mRNA in HeLa cells with two different siRNAs against human *SRSF1*, and found that down regulation of SRSF1 increased usage of the intron-distal 5′ SS in the minigene transcripts as well as in endogenous *DOK7* transcripts (Fig. 4d).

To further demonstrate that direct association of SRSF1 with the identified *cis*-element modulates alternative 5′ SS selection, we artificially tethered SRSF1 to the *cis*-element using MS2-mediated artificial tethering system^34^. First, we made pcDNA-*DOK7-*MS2 minigene, in which the wild-type Block-12 was replaced with the MS2-binding hairpin sequence (Fig. 4e). We also made a cDNA construct expressing SRSF1 fused with the MS2 coat protein (SRSF1-MS2) to artificially tether SRSF1 to the MS2-binding hairpin sequence in pcDNA-*DOK7-*MS2. Western blotting showed efficient expression of SRSF1-MS2 protein in transfected HeLa cells (Fig. 4f). We observed that replacement of the *cis*-element with MS2 hairpin compromised usage of the intron-proximal 5′ SS (Fig. 4g, lane 2), and that tethering of SRSF1-MS2 specifically restored usage of the intron-proximal 5′ SS (Fig. 4g, lane 4). These results indicate that binding of SRSF1 to immediate upstream of the intron-distal 5′ SS shifts the splice site selection from the intron-distal 5′ SS to the intron-proximal 5′ SS.

### Binding of SRSF1 to the *cis*-element suppresses assembly of U1 snRNP on the intron-distal 5′ SS

**Figure 5:**
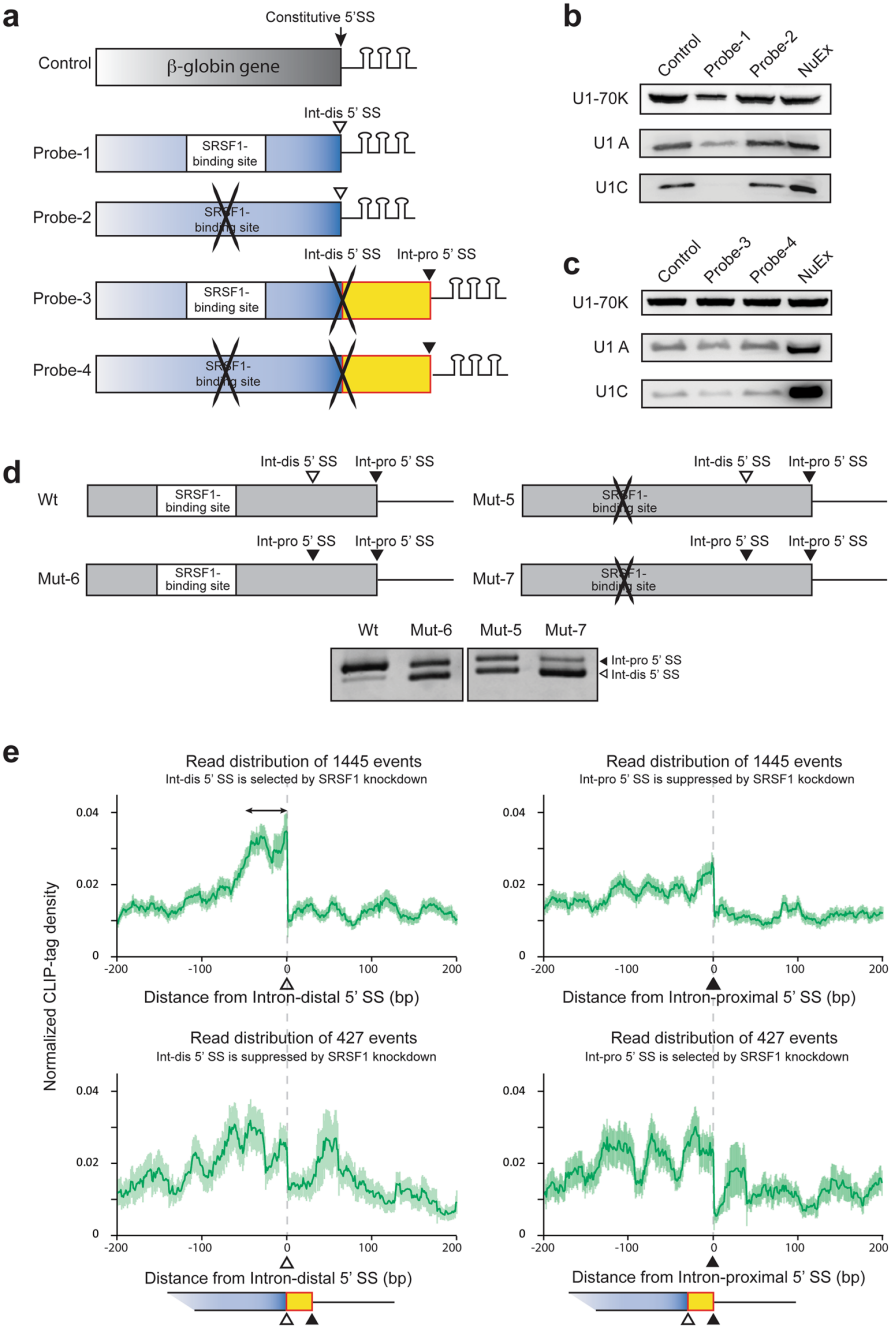
SRSF1 suppresses binding of U1 snRNP to the intron-distal 5′ SS of *DOK7* intron 4. (**a**) Schematic of 3 x MS2 RNA probes used for isolation of an early spliceosome complex. Control probe contains constitutive 5′ SS of the human β-globin gene encoded in pSP64-HβΔ6-MS2. Probe-1 carries the SRSF1-binding site and the intron-distal (Int-dis) 5′ SS, but lacks the intron-proximal (Int-pro) 5′ SS. Probe-2 carries a disrupted SRSF1-binding site and the intron-distal 5′ SS, but lacks the intron-proximal 5′ SS. Probe-3 carries the SRSF1-binding site, a mutated intron-distal 5′ SS, and the intron-proximal 5′ SS. Probe-4 carries a disrupted SRSF1-binding site, a mutated intron-distal 5′ SS, and the intron-proximal 5′ SS. Sequences of the probes are shown in Supplementary Fig. S3. (**b**) Immunoblotting of purified spliceosome complexes assembled on control, probe-1, and probe-2 RNA substrates. (**c**) Immunoblotting of purified spliceosome complexes assembled on control, probe-3, and probe-4 RNA substrates. (**d**) Schematic of *DOK7* minigenes carrying wild-type (Wt and Mut-6) or disrupted (Mut-5 and Mut-7) SRSF1-binding site, with (Mut-6 and Mut-7) or without (Wt and Mut-5) duplicated intron-proximal 5′ SS. Lower panel shows RT-PCR of these minigenes transfected in HeLa cells. Sequences of the Wt and mutant constructs (Mut-5, -6 and -7) are shown in Supplementary Fig. S6. Mut-5 is identical to Mut-5 in Fig. 3d. (**e**) Distribution of SRSF1-CLIP tags centered around the intron-distal (left panels) and intron-proximal (right panels) 5′ SSs depicted by integrated genome-wide analysis of CLIP-seq of SRSF1 in native HeLa cells and RNA-seq in *SRSF1*-knocked down HeLa cells. Mean (green lines) and standard error (light green areas) of normalized CLIP-tag densities are shown. In 1445 (upper panels) and 427 (lower panels) genes, SRSF1-konockdown activates intron-distal and intron-proximal 5′ SSs, respectively. A double-headed arrow indicates a peak immediately upstream to the intron-distal 5′ SS in the 1445 genes, indicating the suppressive effect of SRSF1 on the intron-distal 5′ SS, as we observed in *DOK7* intron 4.

To dissect the underlying mechanisms of how binding of SRSF1 immediately upstream to the intron-distal 5′ SSs regulates alternative 5′ SS selection, we examined the effect of SRSF1 on the assembly of U1 snRNP to either of these 5′ SSs. We first made two 3 x MS2-attached RNA probes (Fig. 5a and Supplementary Fig. S3); Probe-1 contained the intron-distal 5′ SS with the SRSF1-binding site, whereas probe-2 contained the intron-distal 5′ SS with a disrupted SRSF1-binding site. As a control, we used the 3 x MS2-attached human β-globin gene^35^ (Fig. 5a and Supplementary Fig. S3). We added these 3 x MS2-attached RNA probes to the splicing-competent HeLa nuclear extract to make an early spliceosome assemble on the probe. Spliceosome on each probe was isolated using the MS2 coat protein-coated beads, and Western blot analysis was performed as previously described^35^. We found that association of U1 snRNP to probe-1, but not to probe-2, was markedly reduced compared to the control probe (Fig. 5b, lanes 2 and 3), suggesting that binding of SRSF1 to the *cis*-element has a suppressive effect on the assembly of U1 snRNP on the adjacent intron-distal 5′ SS. We next analyzed the effect of SRSF1 on the assembly of U1 snRNP on the intron-proximal 5′ SS. We made two additional 3 x MS2-attached RNA probes; Probe-3 contained the intron-proximal 5′ SS with the SRSF1-binding site, and probe-4 contained the intron-proximal 5′ SS with a disrupted SRSF1-binding site (Fig. 5a and Supplementary Fig. S3). The intron-distal 5′ SS was mutated in both probes-3 and -4. Analysis of the associated complexes on these probes showed that the assembly of U1 snRNP on the intron-proximal 5′ SS was not affected by the disruption of the SRSF1-binding site (Fig. 5c), which was in contrast to the effect on the intron-distal 5′ SS (Fig. 5b).

We next made additional minigenes, where the intron-distal 5′ SSs was replaced with the intron-proximal 5′ SS (Mut-6 and Mut-7 in Fig. 5d). Substitution of the intron-proximal 5′ SS (Mut-6) for the intron-distal 5′ SS (Wt) compromised the suppressive effect of SRSF1 (Fig. 5d, lanes 1 and 2). This was likely due to a stronger splicing signal of the intron-proximal 5′ SS than that of the intron-distal 5′ SS. When the SRSF1-binding site was disrupted (Mut-5 and Mut-7), selection of the upstream 5′ SS was more enhanced (Fig. 5d, lanes 3 and 4). This indicates that SRSF1 is able to suppress the selection of an adjacent 5′ SS independent of its sequence context.

### Binding of SRSF1 to a *cis*-element immediately upstream to tandem 5′ SSs suppresses selection of the intron-distal 5′ SS in other genes

**Figure 6:**
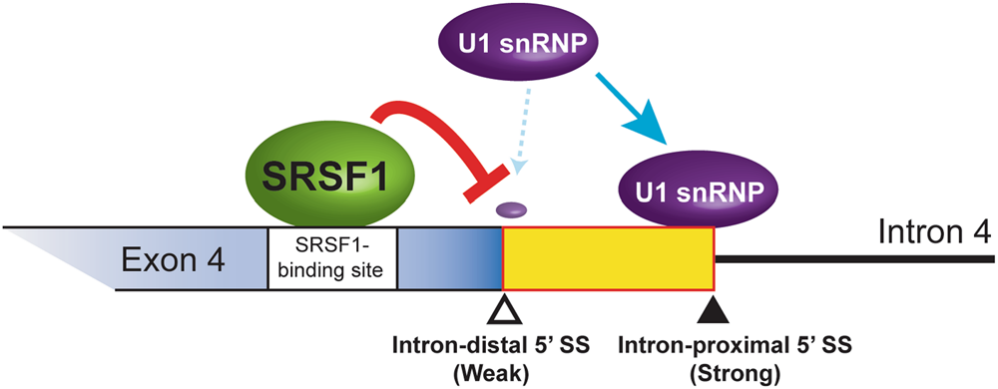
Schematic of alternative 5′ SS selection of human *DOK7* intron 4. Binding of SRSF1 immediately upstream to the intron-distal 5′ SS suppresses binding of U1 snRNP to the intron-distal 5′ SS, but not to the intron-proximal 5′ SS.

We have shown that binding of SRSF1 immediately upstream to tandem 5′ SSs suppresses selection of the adjacent intron-distal 5′ SS in the human *DOK7* gene. To examine whether what we observed with *DOK7* intron 4 is applicable to other genes in the human genome, we analyzed RNA-seq of *SRSF1*-knocked down HeLa cells (GSE26463) and CLIP-seq of SRSF1 in HeLa cells (GSE71096), which were deposited in the GEO database^36, 37^. Splicing analysis of RNA-seq with MISO^38^ detected 1445 and 427 alternative 5′ splicing events, in which the intron-distal 5′ SS and the intron-proximal 5′ SS were selected by knockdown of *SRSF1*, respectively (Fig. 5e). SRSF1-regulated alternative splicing of *DOK7* intron 4 is similar to the 1445 alternative 5′ splicing events, in which the intron-distal 5′ SS was selected by SRSF1 knockdown. We analyzed the distribution of SRSF1-CLIP tags around these alternative 5′ SSs. We found that SRSF1 clustered immediately upstream to the intron-distal 5′ SS in 1445 exons (double-headed arrow in Fig. 5e, upper left panel), which was selected by *SRSF1*-knockdown. In contrast, no noticeable SRSF1 cluster was observed around the intron-proximal 5′ SS in these 1445 exons (Fig. 5e, upper right panel). To further dissect SRSF1-CLIP tag coverage on the 1445 exons at 50-nt resolution, we divided 400 nt region spanning the 5′ SS into eight 50-nt sections (Supplementary Fig. S4a, upper panel), and analyzed which 50-nt section has the highest SRSF1-CLIP tag coverage. We found that the highest SRSF1-CLIP tag coverage was most frequently observed (309 intron-distal 5′ SSs) immediately upstream to the 5′ SS (section 4 in Supplementary Fig. S4a and Supplementary Data S1). Three representative 5′ SSs are also indicated in Supplementary Fig. S4b. Although we observed less conspicuous binding of SRSF1 around both the intron-distal and intron-proximal 5′ SSs in the 427 genes, where knockdown of *SRSF1* enhanced selection of the intron-proximal 5′ SS (Fig. 5e, lower panels), there was no noticeable difference between the intron-distal and intron-proximal 5′ SSs. Thus, the functional significance of SRSF1-binding on these 427 genes remains to be determined. To summarize, binding of SRSF1 immediately upstream to the intron-distal 5′ SS suppresses selection of the intron-distal 5′ SS in many human genes (Fig. 6).

## Discussion

We analyzed the regulatory mechanisms of alternative selection of two 5′ SSs at intron 4 of *DOK7*, which encodes an indispensable adaptor protein for enhancing AChR clustering at the neuromuscular junction^23^. These 5′ SSs are only 11 nucleotides apart, and their sequences are well conserved across mammalian species (Supplementary Fig. S5a). Although alternative splicing in non-human species have not been reported or annotated, selection of the intron-distal 5′ SS causes a frame-shift in *DOK7* mRNA with a premature termination codon (PTC) in all of these species (Supplementary Fig. S5b). This PTC-harboring *DOK7* transcript constitutes around a quarter of *DOK7* transcripts in the skeletal muscle, brain, and heart in human, where *DOK7* is highly expressed (Fig. 1b). The truncated Dok-7 isoform 2 marginally enhanced MuSK phosphorylation (Fig. 2c), but significantly diminished the AChR clustering activity (Fig. 2a,b).

The canonical full-length Dok-7 isoform 1 carries pleckstrin-homology (PH) and phosphotyrosine-binding (PTB) domains in the N-terminal region, as well as two tyrosine residues, which are target motifs of the Src homology 2 (SH2) domain, in the C-terminal region^23^ (Fig. 1a). The PH domain is essential for membrane association, and PTB domain is involved in Dok-7-induced activation of MuSK^39^. Activated MuSK phosphorylates the two tyrosine residues (Y396 and Y406) of Dok-7, which are essential for activating downstream signaling leading to AChR clustering^26^. Preservation of the PH domain and part of the PTB domain in Dok-7 isoform 2 (Fig. 1a) may account for minimal induction of MuSK phosphorylation in C2C12 myotubes overexpressing the truncated Dok-7 isoform 2 (Fig. 2c). Lack of the tyrosine residues, however, is likely to have abolished AChR clustering activity of the truncated Dok-7 isoform (Fig. 2a,b).

We have identified SRSF1 as a regulator of selection of two 5′ SSs of *DOK7* intron 4. Binding of SRSF1 immediately upstream to the two competing 5′ SSs suppresses selection of the intron-distal 5′ SS. SRSF1 is a member of serine and arginine-rich (SR) protein family, which regulates multiple steps of RNA processing, including pre-mRNA splicing^20^, transcription, mRNA stability, nuclear export, NMD, and protein translation^21^. In contrast to our finding, a previous study showed that artificial tethering of SRSF1 between two competing 5′ SSs facilitates use of the intron-proximal 5′ SS^17^. Thus, SRSF1 may exert the opposing effects on alternative 5′ SS selection in a position-specific manner. We showed by an integrated global analysis of CLIP-seq of SRSF1 and RNA-seq of *SRSF1*-knocked down cells that binding of SRSF1 immediately upstream to two competing 5′ SSs generally suppresses the intron-distal 5′ SS (double-headed arrow in Fig. 5e). In contrast, facilitation of the intron-proximal 5′ SS by binding of SRSF1 between two competing 5′ SSs reported by others^17^ was not distinctly corroborated in our integrated analysis.

Isolation of an early spliceosome complex revealed that SRSF1 inhibits binding of U1 snRNP to the intron-distal 5′ SS to suppress selection of the intron-distal 5′ SS of *DOK7* intron 4. In contrast to our study, a previous report shows that SRSF1 directly interacts with U1 snRNP to make an early spliceosome complex and facilitates splicing^40^. In this report^40^, SRSF1 bridges pre-mRNA to U1-70K, a specific component of U1 snRNP, through their RRMs. Contrarily, the SRSF1-bidning *cis*-element and the intron-distal 5′ SS are adjacently located in *DOK7*, which may sterically hinder simultaneous binding of SRSF1 and U1-70K snRNP to closely located RNA segments. In contrast to the intron-distal 5′ SS, we found that SRSF1 has no effect on binding of U1 snRNP to the intron-proximal 5′ SS (Fig. 5c). This can be accounted for by two possible mechanisms. First, as placement of the intron-proximal 5′ SS to the position of the intron-distal 5′ SS made the minigene construct sensitive to the suppressive effect of SRSF1 (Mut-6 and Mut-7 in Fig. 5d), the intron-proximal 5′ SS was immune to SRSF1 because of the distance from the SRSF1-binding *cis*-element. Second, the intron-proximal 5′ SS has a higher splicing strength than the intron-distal 5′ SS (Fig. 1a). Especially, intronic sequence of “gtaagt” of the intron-proximal 5′ SS is complementary to the 5′ end of U1 snRNA that binds to the pre m-RNA^41^. We conclude that alternative selection of the two 5′ SSs of *DOK7* intron 4 is finely tuned by (i) binding of SRSF1 to their immediate upstream position, (ii) the distance between the SRSF1-binding site and 5′ SS, and (iii) the strength of splicing signals of the two 5′ SSs. Similar splicing regulations are likely to be operational in many other human genes.

## Methods

### Cell culture and transfection

HeLa and C2C12 cells were cultured in the Dulbecco’s minimum essential medium (DMEM, Sigma-Aldrich) supplemented with 10% and 20% fetal bovine serum (Sigma-Aldrich), respectively. Immortalized KD3 human myoblasts were kindly provided by Dr. Naohiro Hashimoto at National Center for Geriatrics and Gerontology, Japan^42^. KD3 cells were grown in high-glucose (4.5 g/ml) DMEM (hDMEM) medium containing 20% FCS and 2% Ultroser G serum substitute (PALL), as previously described^43^. To induce myogenic differentiation of confluent C2C12 myoblasts, the culture medium was switched to DMEM supplemented with 2% horse serum and 1x Insulin-Transferrin-Selenium (Thermo Fisher Scientific). HeLa cells were transfected with FuGENE 6 (Roche), and KD3 and C2C12 cells were transfected with Lipofectamine 3000 (Thermo Fisher Scientific) according to the manufacturers’ protocols.

### Construction of minigenes

Exons 3, 4, and 5, and their flanking intronic 150 nucleotides of human *DOK7* were amplified by a proofreading DNA polymerase (PrimeSTAR, Takara) using DNA extracted from KD3 cells. The three PCR products were comprised of (i) exon 3 to intron 3 (IVS3+150); (ii) intron 3 (IVS3-150) to intron 4 (IVS4+150); and (iii) intron 4 (IVS4 −150) to the first 20 nucleotides of exon 5. As the sizes of introns 3 and 4 were 2705 and 8996 nucleotides, 2405 and 8696 nucleotides in the middle of introns 3 and 4 were excluded from amplification, respectively. PCR primers additionally carried a restriction site at their 5′ end that matched to the neighboring amplicon. Primer sequences and restriction sites are shown in Supplementary Table S1. The PCR products were digested by restriction enzymes, and ligated using DNA ligation kit (Takara). The ligated product was amplified again by PCR, digested by restriction enzymes, and cloned into pcDNA3.1+ vector (Fig. 3a).

Block scanning mutagenesis, site-directed mutagenesis to make Mut-1 to Mut-7 constructs, introduction of MS2-binding hairpin sequence, and placement of the intron-proximal 5′ SS were performed with the QuikChange site-directed mutagenesis kit (Agilent) with oligonucleotides indicated in Supplementary Table S1. All constructs were sequenced to ensure the presence of desired mutation(s) and the absence of unexpected artifacts.

### Construction of expression vectors

cDNA for human *DOK7* transcript variant 1 was amplified from total RNA of KD3 cells, and cloned into the pEGFP-N1 expression vector (Clontech) to make pEGFP-*DOK7*-T-var1. pEGFP-*DOK7*-T-var2 to express a fusion protein comprised of Dok-7 isoform 2 and EGFP was made from pEGFP-*DOK7*-T-var1 by deleting the 11 nucleotides at the 3′ end of exon 4, as well as a premature termination codon and its downstream nucleotides of *DOK7*. Construction of expression vectors for human FLAG-MuSK^44^, SRSF1^45^, SRSF1-MS2^45^ and hnRNP H-MS2^46^ were previously reported. The absence of artifacts was confirmed by sequencing the entire insert.

### Transfection of minigenes and trans-acting factors

HeLa cells were plated 24 h prior to transfection in a six-well culture plate (1.5 × 10^5^ cells/well) and transfected with 500 ng of the pcDNA-human-*DOK7* minigene or pcDNA-human-*DOK7*-MS2 minigene, as well as with or without 1 µg of an expression vector for SRSF1, SRSF1-MS2, or hnRNP H-MS2, using 6.0 μl FuGENE 6 (Roche) in 100 μl Opti-MEM medium according to the manufacturer’s instructions.

### RT-PCR

Total RNA was extracted 48 h after transfection using Trizol (Invitrogen), followed by DNase I treatment. cDNA was synthesized with oligo-dT primer (Invitrogen) for RT-PCR and random primer (Invitrogen) for Real-time RT-PCR using ReverTra Ace reverse transcriptase (Toyobo), and RT-PCR was performed with GoTaq (Promega) using primers shown in Supplementary Table S2.

Real-time RT-PCR was performed using LightCycler 480 II (Roche) and the SYBR Premix Ex Taq II (Takara) to quantify endogenous human *SRSF1* transcripts using primers shown in Supplementary Table S3.

### RNA affinity purification assay

Biotinylated RNA probes were synthesized with T7 RiboMAX large-scale RNA production system (Promega) using DNA templates generated by hybridizing two complementary oligonucleotides shown in Supplementary Table S4. RNA affinity purification was performed as previously described^35^ with some modifications. Briefly, biotinylated RNA (0.75 nmol) and HeLa nuclear extract (30 μl) (CilBiotech) were mixed in a 500-μl binding buffer [20 mM HEPES, pH 7.8, 125 mM KCl, 0.1 mM EDTA, 1 mM DTT, 1 mM PMSF, 0.05% Triton X, 1× Protease Inhibitor Cocktail (Active Motif)], and were incubated at 30 °C for 3 h with gentle agitation. In parallel, 50 μl streptavidin-conjugated beads (Streptavidin-sepharose, GE Healthcare) were blocked with a 1:1 mixture of 1 ml binding buffer containing yeast tRNA (0.1 mg/100 μl of beads) and 1 ml PBS containing 4% BSA at 4 °C with rotation for 1 h. The beads were washed four times and incubated with the binding buffer at 30 °C for 1 h with gentle rotation. After washing the beads four times with 1 ml binding buffer, RNA-bound proteins were eluted in SDS loading buffer by boiling at 95 °C for 5 min. The isolated proteins were fractionated on a 12% SDS-polyacrylamide gel and stained with Coomassie blue or by immunoblotting.

### Mass spectrometry

Mass spectrometry analysis of affinity purified RNA binding proteins was performed as previously described^35^. Briefly, a Coomassie blue-stained band of interest was excised from the gel and was digested in-gel by Trypsin Gold (Promega) according to the manufacturer’s protocols. For in-solution digestion, the RNA-bound proteins were eluted in an elution buffer (0.1 M glycine with 2 M urea, pH 2.9) and digested by Trypsin Gold according to the manufacturer’s recommendations. Nanoelectrospray tandem mass analysis was performed using an LCQ Advantage Mass Spectrometry System (Thermo Finnigan). Multiple MS/MS spectra were analyzed by the Mascot program version 2.4.1 (Matrix Science)^35^.

### Constructs for an early spliceosomal complex assay

To isolate an early spliceosomal complex, three copies of MS2 hairpin sequences (3x MS2) were transferred from pSP64-MS2^35^ to pcDNA3.1+. We then introduced wild-type *DOK7* sequence including the SRSF1-binding *cis*-element and the intron-distal 5′ SS into pcDNA3.1-MS2 by QuikChange site-directed mutagenesis kit (Stratagene) to make a construct to generate probe-1. We introduced mutations into the construct for probe-1 using the QuikChange method to make constructs for probes-2, -3, and -4. Sequences of probes-1, -2, -3, and -4 are shown in Supplementary Fig. S3. RNA probes were generated by the T7 RiboMAX large-scale RNA production system (Promega). As control, we used MS2-attached human β-globin construct (pSP64-HβΔ6-MS2) as previously described^35^.

### MS2-affinity isolation of an early spliceosomal complex

One pmol of RNA probe (a control transcript from pSP64-HβΔ6-MS2, and probes-1 to -4) with 20-fold molar excess of MS2-MBP fusion protein^47^ was incubated in a 200-µl binding buffer [20 mM HEPES pH 7.8, 60 mM KCl, 0.5 mM EDTA, 20% glycerol, 1 mM DTT, 1 mM PMSF, and 1× Protease Inhibitor Cocktail (Active Motif)] overnight at 4 °C with gentle agitation. In parallel, 50 μl of HeLa nuclear extract (CILBiotech) was pretreated with 10 μl (bead volume) of amylose resin (New England Biolabs) in a 200-µl binding buffer. The RNA probe with MS2-MBP fusion protein in 200 µl was then added with 50 µl of the pretreated HeLa nuclear extract, as well as with a 200-μl binding buffer. The ~450-µl mixure was incubated at 37 °C for 30 min. Amylose resin beads (20 μl) were then added in the mixture, and incubated at 4 °C for 2 h with gentle rotation. After washing the resin four times with a washing buffer (20 mM HEPES pH 8.0, 10 mM MgCl_2_, 100 mM KCl, 0.01% NP-40, and 1 mM DTT), bound proteins were eluted with 10 mM maltose and subjected to SDS-PAGE for Western blotting.

### siRNA knockdown and minigene splicing

We synthesized two human siRNAs (Sigma Genosys) for *SRSF1*-knockdown. The sequences of the two siRNAs were 5′-CCAAGGACAUUGAGGACGUTT-3′ and 5′-GGAAAGAAGATATGACCTATT-3′. The control siRNA was AllStar Negative Control siRNA (1027281) by Qiagen. Cells were plated 24 h before transfection in a six-well culture plate (1.5 × 10^5^ cells/well). Each siRNA duplex at a final concentration of 30 nM was mixed with 7.5 μl Lipofectamine RNAiMAX (Thermo Fisher Scientific) in 100 μl Opti-MEM medium. For splicing analysis of a minigene, 500 ng minigene and 6.0 µl of FuGENE 6 were mixed with 100 µl Opti-MEM medium, and add to the culture medium at 24 h after transfection of siRNA. The cells were harvested at 48 h after transfection of siRNA for RT-PCR and Western blotting.

### Antibodies

Antibodies used in this study were anti-SRSF1 (dilution 1:1000, 32–4500, Invitrogen), anti-U1-70K (dilution 1:1000, H111, kindly provided by Dr. Akila Mayeda at Fujita Health University), anti-U1A (dilution 1:1000, PA5-27474, Thermo Fisher Scientific), anti-U1C (dilution 1:1000, SAB4200188, Sigma-Aldrich), anti-His-tag (dilution 1:2000, D291-3, Medical & Biological Laboratories), anti-FLAG (dilution 1:4000, F1804, Sigma), anti-phosphotyrosine (1 µg for each immunoprecipitation assay, 05–321, EMD MILLIPORE), anti-GFP (dilution 1:1000, 11814460001, Roche), and anti-GAPDH (dilution 1:2500, G9545, Sigma-Aldrich).

### Harvesting cells for immunoblotting

Total cell lysates were harvested using buffer A (10 mM HEPES-NaOH pH 7.8, 10 mM KCl, 0.1 mM EDTA, 1 mM DTT, 0.5 mM PMSF, 0.1% Nonidet P-40, 1× Protease Inhibitor Cocktail). Western blotting was performed as previously described^43^.

### AChR clustering analysis

C2C12 cells were cultured in a six-well culture plate for 24 h, and transfected with an empty pEGFP-N1 vector, pEGFP-*DOK7*-T-var1, or pEGFP-*DOK7*-T-var2. To induce myogenic differentiation of C2C12 cells, the culture medium of confluent cells was switched to DMEM supplemented with 2% horse serum and 1 x Insulin-Transferrin-Selenium (Thermo Fisher Scientific) at 24 h after transfection. After myotube formation, cells were treated with 10 ng/ml agrin to induce AChR clustering for 12 h. Cells were stained with 10 mg/ml Axexa594-conjugated α-bungarotoxin (1:100, Invitrogen) to label AChR and fixed in 2% paraformaldehyde. Fluorescence images were taken under an Olympus XL71 fluorescence microscope and analyzed with MetaMorph software (Molecular Devices).

### MuSK phosphorylation assay

C2C12 cells were cultured in a 3.5-cm cell culture plate coated with collagen I (BD Biosciences). The cells were co-transfected with 3 µg of an expression vector for FLAG-MuSK and 3 µg of pEGFP-*DOK7*-T-var1 or pEGFP-*DOK7*-T-var2, and cultured for 48 h. The cells were lysed with a buffer containing 50 mM HEPES pH 7.0, 150 mM NaCl, 10% glycerol, 1% TritonX-100, 1.5 mM MgCl_2_, 1 mM EGTA, 100 mM NaF, 10 mM sodium pyrophosphate, 1 μg/μl aprotinin, 1 μg/μl leupeptin, 1 μg/μl pepstatin A, 1 mM PMSF, 1 mM sodium orthovanadate, and the PhosSTOP phosphatase inhibitor cocktail (Roche Life Sciences). Cell lysates were immunoprecipitated using 1 µg anti-phosphotyrosine antibody. Immunoprecipitated molecules were detected by Western blotting using anti FLAG antibody.

### RNA-seq analysis

RNA-seq of *SRSF1*-knocked down HeLa cells was obtained from the GEO database (accession number, GSE26463)^36^. The FASTQ sequences were processed by the following procedures: (i) quality-check by FastQC version 0.10.1 (http://www.bioinformatics.babraham.ac.uk/projects/fastqc/), (ii) mapping to UCSC hg19^48^ by TopHat version 2.0.12^49^, (iii) assembly by Cufflinks version 2.2.1^50^ with Ensembl Homo_sapiens.GRCh37.75.gtf ^51^ as guide transcripts. We next detected alternative 5′ splice sites (A5SSs), and computed percent-spliced-in (PSI) of A5SSs by MISO version 0.5.2 with filtering parameters of–num_inc 1–num-exc 1–delta-psi 0.2–bayes-factor 10^38^.

### PAR-CLIP analysis

PAR-CLIP-seq of SRSF1 was obtained from the GEO database (accession number, GSE71096)^37^. The FASTQ sequences were processed by the following procedures: (i) quality-check by FastQC version 0.10.1, (ii) removal of adapters, polyA at the 3′ end, unreadable nucleotides at the 5′ end, and a short sequence <18 bp, (iii) mapping to UCSC hg19 by STAR version 2.5.3^52^, and (iv) conversion of the created BAM file to a BedGraph file by BEDTools version 2.26.0^53^. Finally, using the BedGraph file, we counted reads mapped on A5SS ± 200 nt by HTSlib version 1.4^54^. The normalized CLIP-tag density is calculated by dividing the CLIP-tag coverage at each position by total coverage of CLIP-tags in a 400-nt segment comprised of 200-nt upstream and 200-nt downstream regions of the 5′ SS.

## Electronic supplementary material

Supplementary file

Dataset 1
