# Supplementary material for: SRSF1 suppresses selection of intron-distal 5′ splice site of DOK7 intron 4 to generate functional full-length Dok-7 protein
Source: Sci Rep. 2017 Sep 5;7:10446. doi: 10.1038/s41598-017-11036-z (PMC5585400; doi:10.1038/s41598-017-11036-z)
Supplement: Supplementary file 1 — Supplementary file [file 41598_2017_11036_MOESM1_ESM.pdf]

# Supplementary Information

## SRSF1 suppresses selection of intron-distal 5' splice site of *DOK7* intron 4 to generate functional full-length Dok-7 protein

**Khalid Bin Ahsan[1], Akio Masuda[1, 2, *], Mohammad Alinoor Rahman[1, 2], Jun-ichi Takeda[1], Mohammad Nazim[1], Bisei Ohkawara[1], Mikako Ito[1], Kinji Ohno[1]**

[1]Division of Neurogenetics, Center for Neurological Diseases and Cancer, Nagoya University Graduate School of Medicine, Nagoya, Aichi, Japan

[2]These authors contributed equally to the paper.

[*]Address correspondence to: Akio Masuda, Division of Neurogenetics, Center for Neurological Diseases and Cancer, Nagoya University Graduate School of Medicine, 65 Tsurumai, Showa-ku, Nagoya 466-8550, Japan

Phone: +81-52-744-2447, Fax: +81-52-744-2449, e-mail: amasuda@med.nagoya-u.ac.jp

Supplementary information includes:

- Supplementary Figures S1-S6
- Full-length blots
- Supplementary Tables S1-S4
- Supplementary Data S1

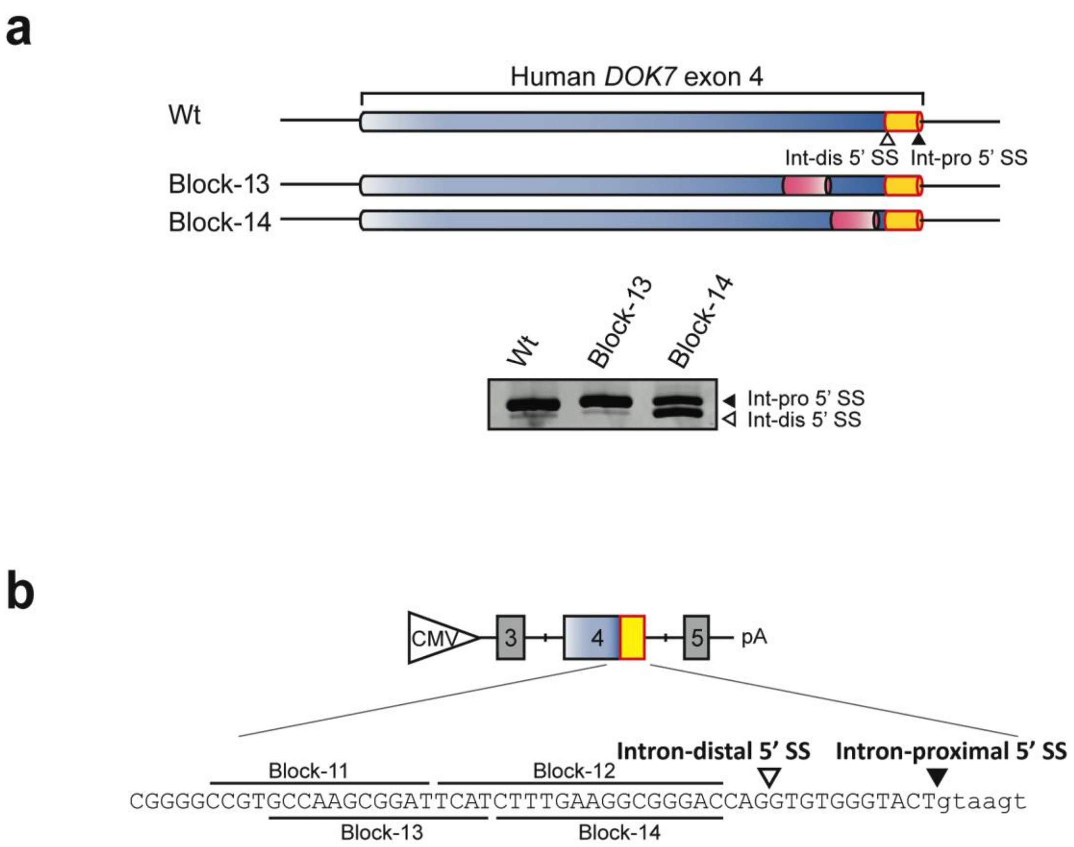


**Supplementary Figure S1.** (a) Schematic of *cis*-regulatory block-scanning mutagenesis of *DOK7* exon 4 from the 3' end in the context of pcDNA-human-*DOK7* minigene. A 15-nucleotide heterologous sequence of 5′-TCAGTATGACTCTCA-3′ is introduced individually into two blocks (Blocks-13, and -14). Positions of intron-distal (Int-dis) and intron-proximal (Int-pro) 5' SS are indicated by open and closed arrowheads, respectively. RT-PCR of these constructs in HeLa cells is shown below. (b) Schematic of *cis*-regulatory block-scanning mutagenesis of *DOK7* exon 4 in the context of pcDNA-human-*DOK7* minigene. A 15-nucleotide heterologous sequence of 5′-TCAGTATGACTCTCA-3′ is introduced into each block, excluding Block-12, which is replaced with 19-nucleotide heterologous sequence of 5′-TCAGTATGACTCTCAGTAT-3′. No mutations are introduced into the first three nucleotides and the last fourteen nucleotides of exon 4, where the intron-distal 5' SS is located. Positions of intron-distal (Int-dis) and intron-proximal (Int-pro) 5' SS are indicated by open and closed arrowheads, respectively. RT-PCR of these constructs in HeLa cells are shown below.

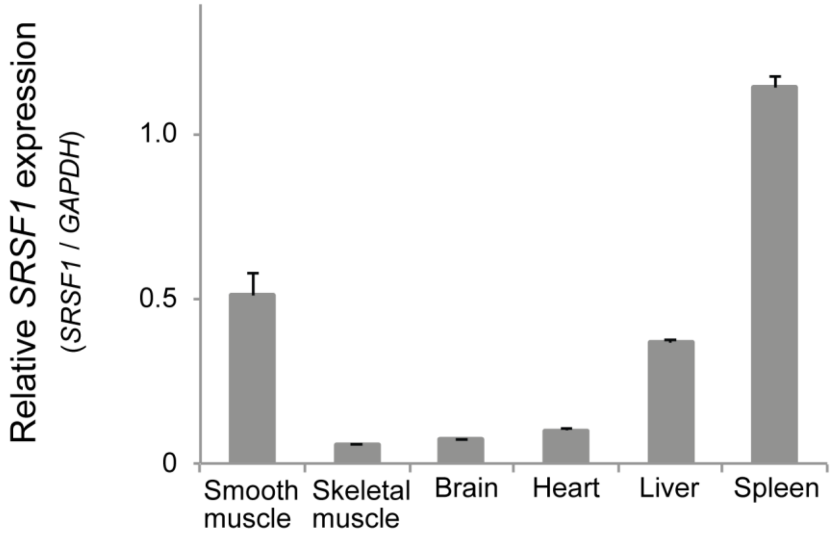


**Supplementary Figure S2.** Real-time RT-PCR to quantify endogenous human *SRSF1* transcripts in different human tissue samples. Transcripts levels are normalized against *GAPDH* values. Bars represent mean and SD of three independent experiments.

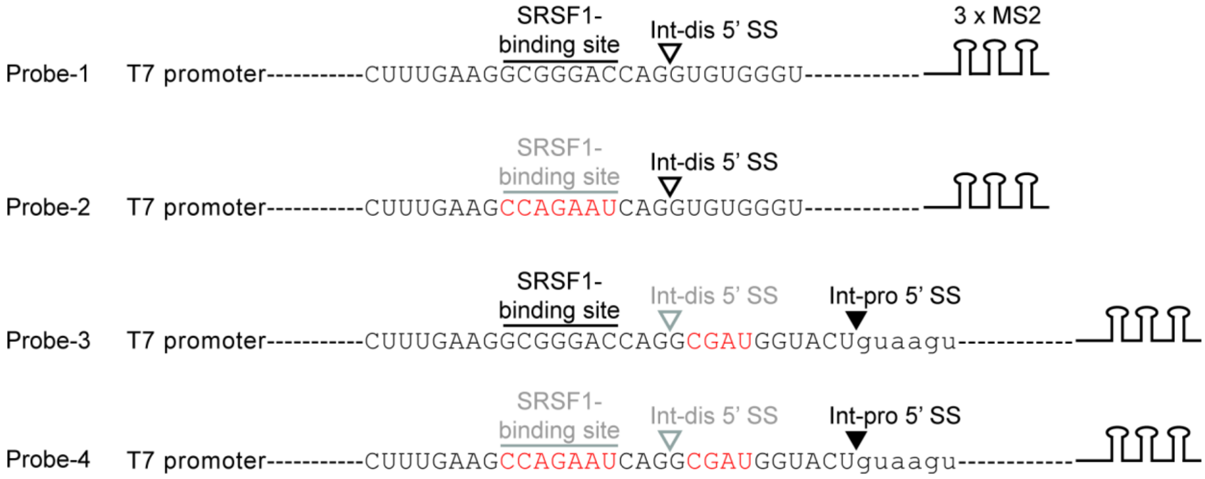


**Supplementary Figure S3.** Nucleotide sequence of RNA probes-1 to -4. Exonic and intronic nucleotides are shown in uppercase and lowercase letters, respectively. Mutant nucleotides are indicated in red. Disrupted elements are indicated in gray.

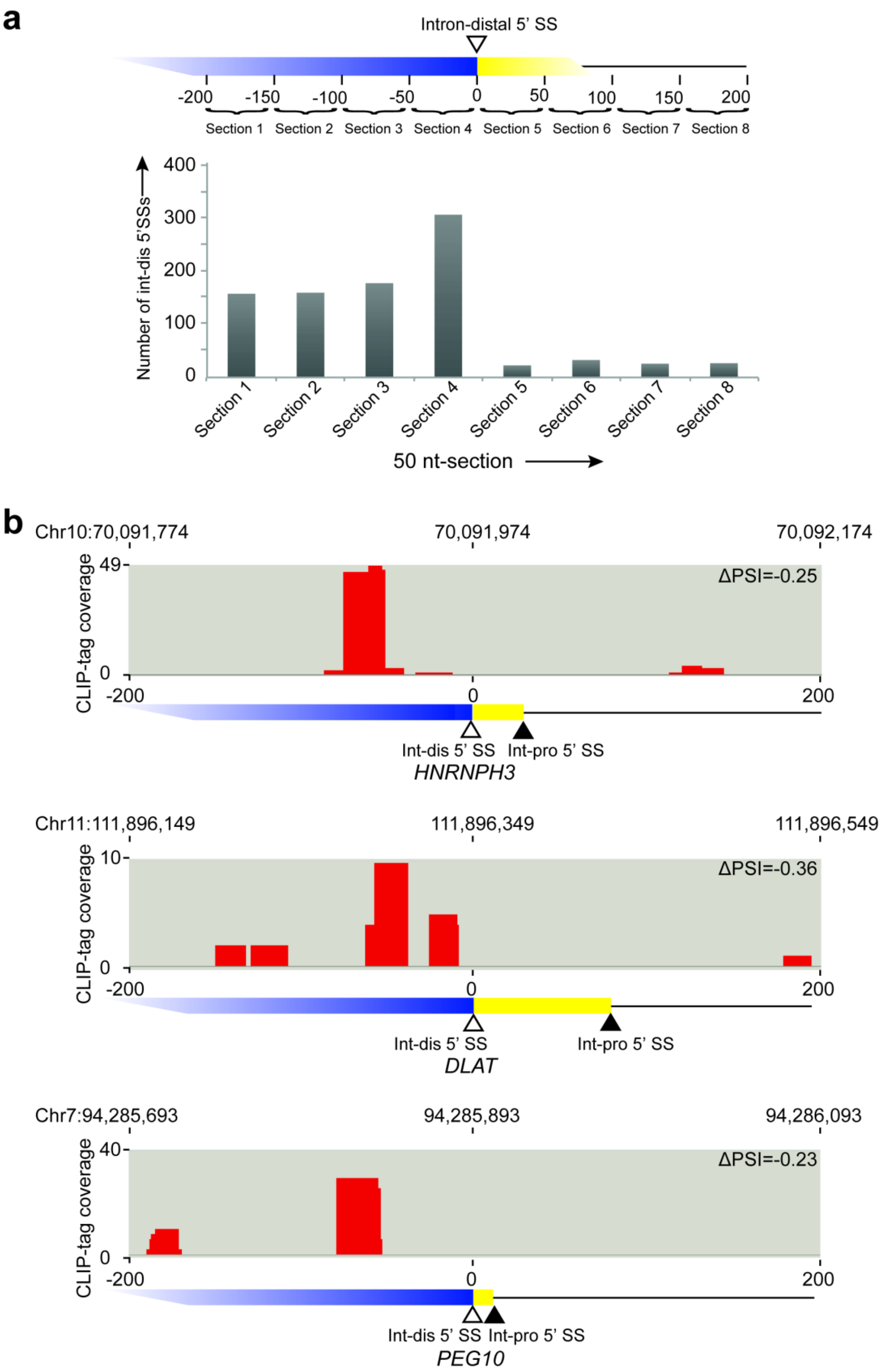
a
Intron-distal 5' SS
-200 -150 -100 -50 0 50 100 150 200
Section 1 Section 2 Section 3 Section 4 Section 5 Section 6 Section 7 Section 8
Number of int-dis 5'SSs
400
300
200
100
0
Section 1 Section 2 Section 3 Section 4 Section 5 Section 6 Section 7 Section 8
50 nt-section
b
Chr10:70,091,774
70,091,974
70,092,174
CLIP-tag coverage
49
0
ΔPSI=-0.25
-200 0 200
Int-dis 5' SS Int-pro 5' SS
HNRNPH3
Chr11:111,896,149
111,896,349
111,896,549
CLIP-tag coverage
10
0
ΔPSI=-0.36
-200 0 200
Int-dis 5' SS
Int-pro 5' SS
DLAT
Chr7:94,285,693
94,285,893
94,286,093
CLIP-tag coverage
40
0
ΔPSI=-0.23
-200 0 200
Int-dis 5' SS Int-pro 5' SS
PEG10

**Supplementary Figure S4.** (a) The upper panel indicates eight 50-nt sections around the intron-distal 5’SS. A section, where the highest SRSF1-CLIP tag coverage is observed, is identified for each of the 1445 5’ SSs, in which SRSF1 knockdown selected the intron-distal 5’ SS. For each section, the number of 5’ SSs with the highest SRSF1-CLIP tag coverage in the particular section is counted, and is plotted in the lower panel. (b) Three representative intron-distal 5’ SSs suppressed by SRSF1 and associated with upstream SRSF1-bindings. Coverages of SRSF1-CLIP tags around the 5’ SSs are shown. ΔPSI indicates the difference in percent-spliced-in (PSI) of the intron-distal 5’ SS between control and *SRSF1*-knocked down cells. Nucleotide coordinates are according to GRCh37/hg19.

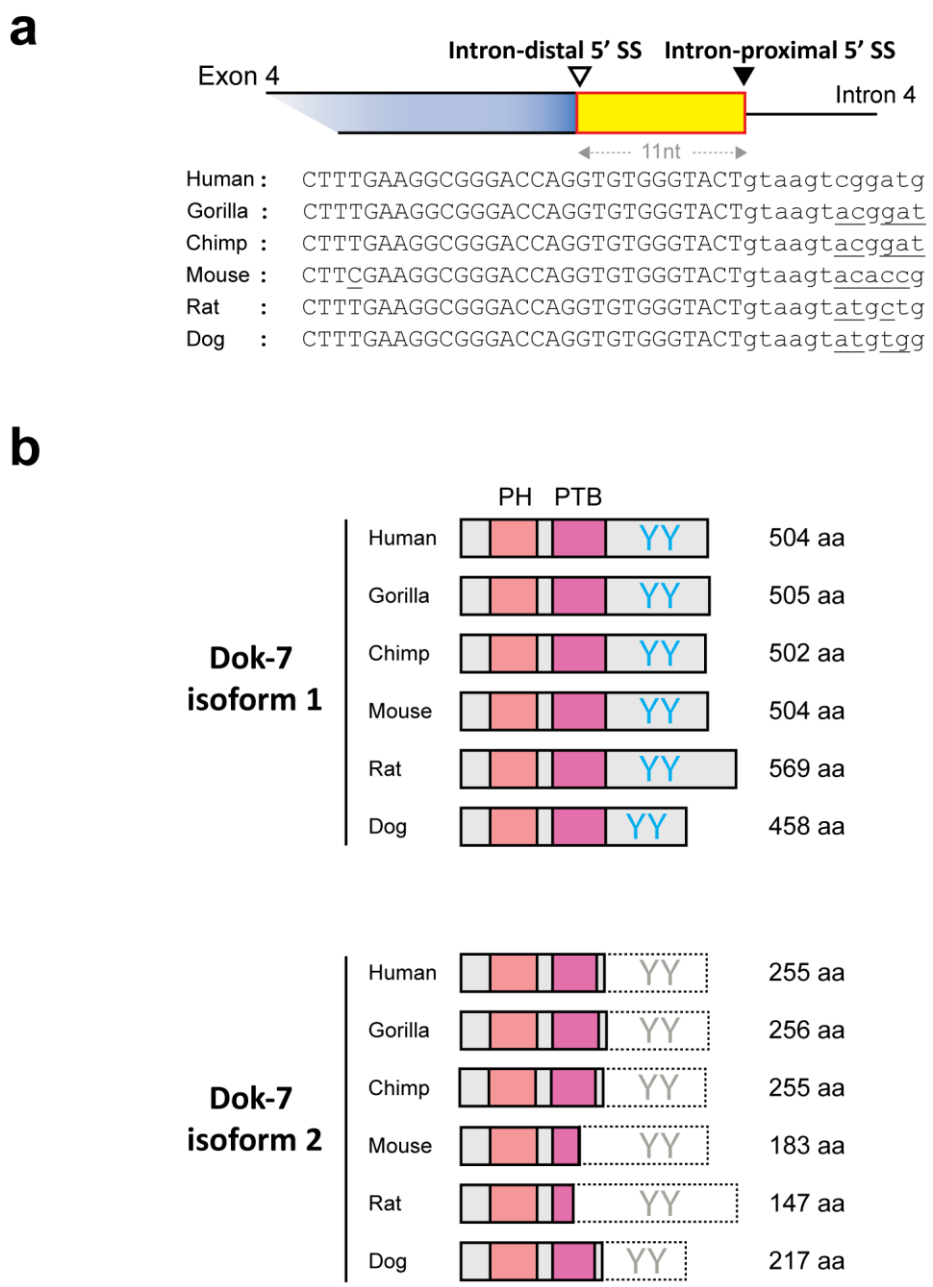


**Supplementary Figure S5.** (a) Genomic sequences around the two 5' SSs of *DOK7* intron 4 in various mammalian species. Exonic and intronic nucleotides are shown in uppercase and lowercase letters, respectively. Different nucleotides compared to human sequence are underlined. (b) Schematic of Dok-7 isoforms 1 and 2 of various mammalian species. Note that isofrom 2 is annotated only in human in the RefSeq, Ensembl, GENCODE, UCSC, AceView, H-Inv, and Vega annotation databases. In all the species, isoforms 1 contain PH domain, PTB domain, and two tyrosine residues, which are indicated by orange boxes, pink boxes, blue YYs, respectively. Truncated regions and lack of the two tyrosine residues in isoforms 2 are indicated by dotted boxes and gray YYs, respectively.

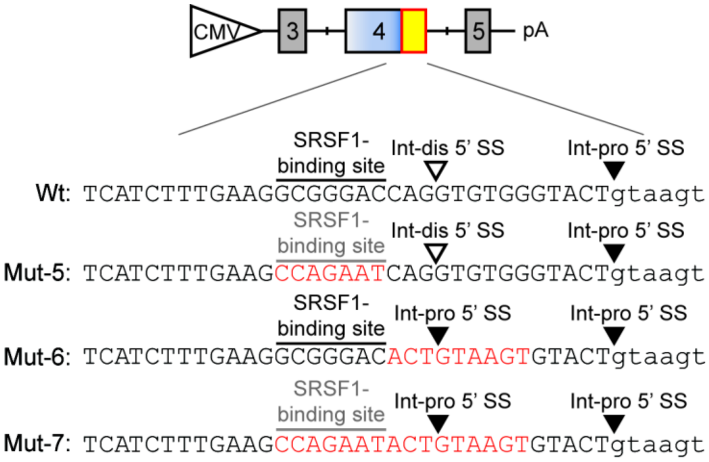


**Supplementary Figure S6.** Schematic of pcDNA-human-*DOK7* minigenes carrying wild-type (Wt) and mutant (Mut-5, -6, and -7) sequence. Exonic and intronic nucleotides are shown in uppercase and lowercase letters, respectively. Mutant nucleotides are indicated in red. Disrupted elements are indicated in gray.

**Full-length blots for main figures**

Figure 2c

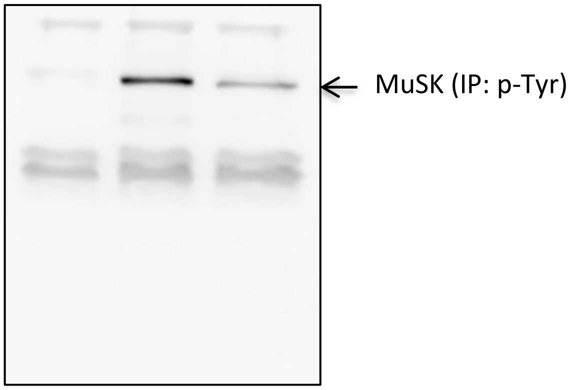


Figure 2c

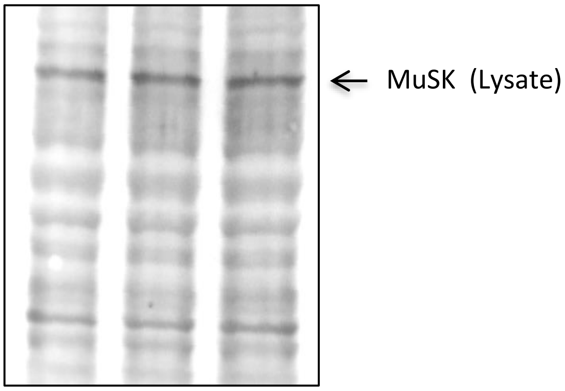


Figure 2c

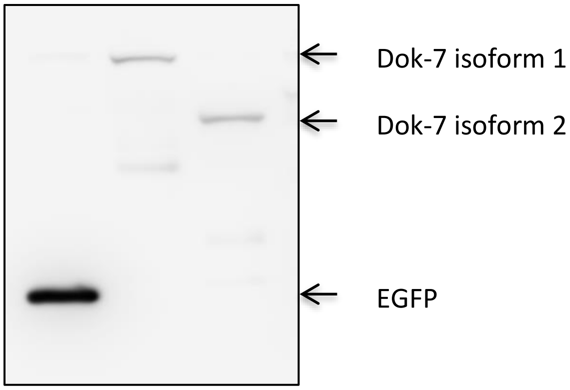


Figure 4c

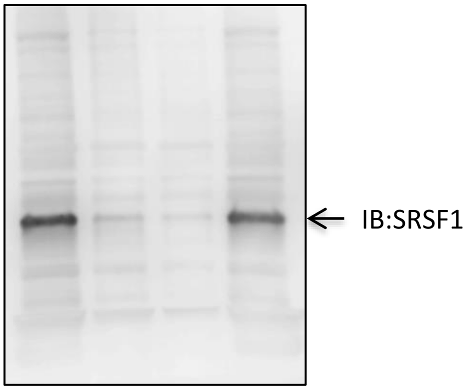


Figure 4d

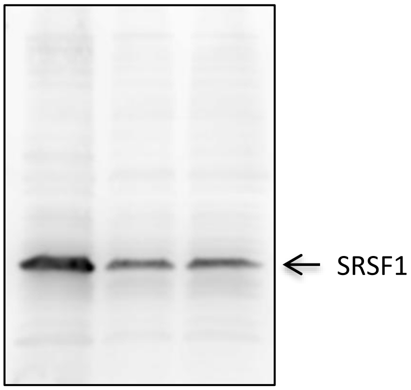


Figure 4d

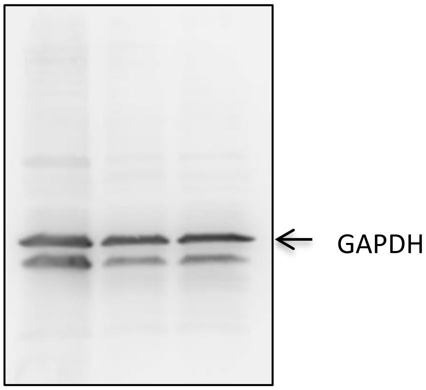


Figure 4f

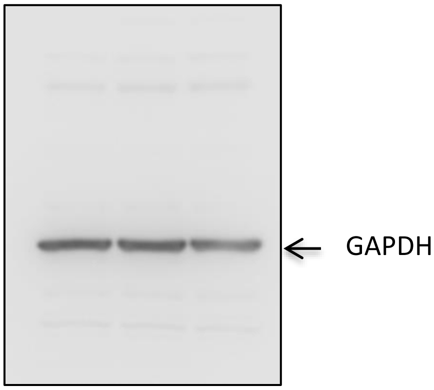

Figure 5b

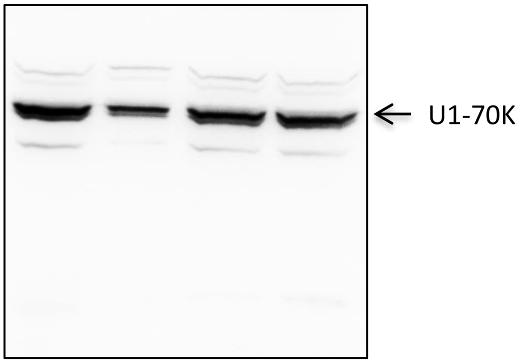


Figure 5b

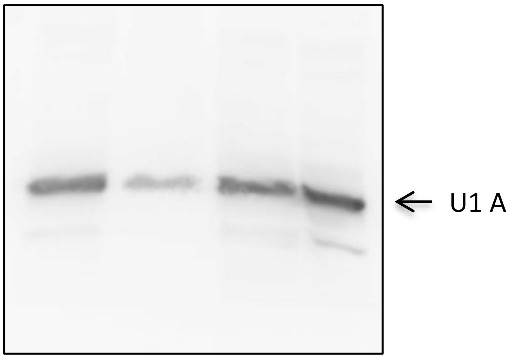


Figure 5b

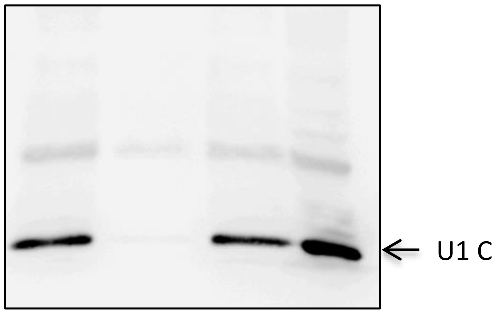


Figure 5c

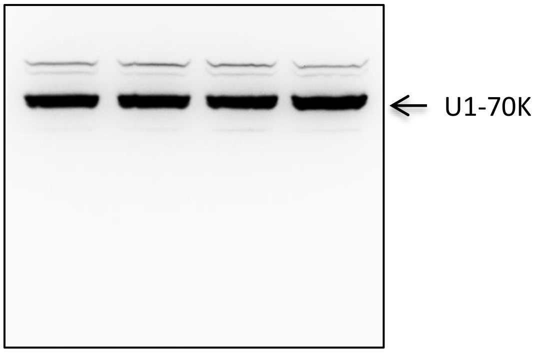


Figure 5c

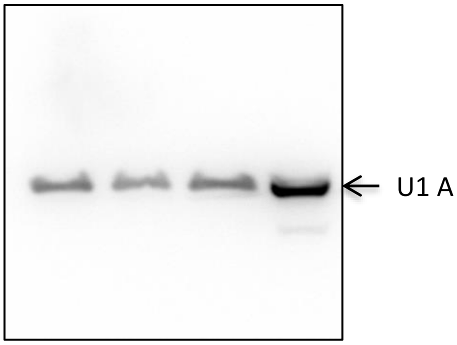


Figure 5c

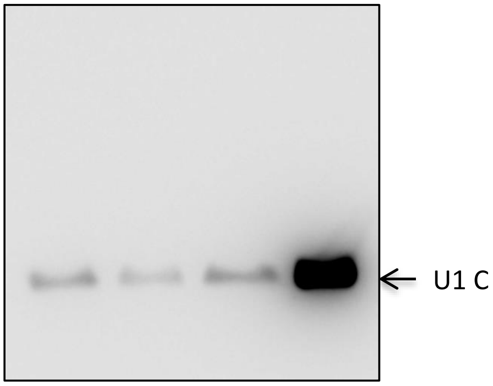

**Supplementary Table S1. PCR primers and oligonucleotides for making constructs**

| Primers | 5'– 3' sequences |
|---|---|
| **Construction of pcDNA-human-*DOK7*** | |
| hDOK7ex3/F | AGTTAAAGCTTCACCATGTGCCTGCTGATGCTGGTCT (HindIII) |
| hDOK7 in3/R | TGACCCTCGAGCTGGAGCTCAGTCCA (XhoI) |
| hDOK7 in3/F | CAGGTCTCGAGCTCACCTCACCCGCCCATGA (XhoI) |
| hDOK7 in4/R | TAGGTGAATTCAGCGTGGGGAGACTCCGTCCA (EcoRI) |
| hDOK7 in4/F | AGAGCGAATTCGTCGGCTCTGGGCATCTGACTTC (EcoRI) |
| hDOK7 ex5/R | GGATATCTAGATCAGCCTAGGCCGAGGACAGGAAGAAGA (XbaI) |
| **Block scanning mutagenesis (QuikChange method)** | |
| D7BM1/F | GCCTGCCCGCAGTGCTCAGTATGACTCTCACAGTGGCTCCAGGCA |
| D7BM1/R | TGCCTGGAGCCACTGTGAGAGTCATACTGAGCACTGCGGGCAGGC |
| D7BM2/F | ATAGGTTCCATGTGATCAGTATGACTCTCACCAAGTTGGAGAGCG |
| D7BM2/R | CGCTCTCCAACTTGGTGAGAGTCATACTGATCACATGGAACCTAT |
| D7BM3/F | CAGTGGCTCCAGGCATCAGTATGACTCTCAGCCCGGCTACCCTGC |
| D7BM3/R | GCAGGGTAGCCGGGCTGAGAGTCATACTGATGCCTGGAGCCACTG |
| D7BM4/F | CCAAGTTGGAGAGCGTCAGTATGACTCTCAACCTCTGCAATGATG |
| D7BM4/R | CATCATTGCAGAGGTTGAGAGTCATACTGACGCTCTCCAACTTGG |
| D7BM5/F | GCCCGGCTACCCTGCTCAGTATGACTCTCATCCTCGTCTTGGCCA |
| D7BM5/R | TGGCCAAGACGAGGATGAGAGTCATACTGAGCAGGGTAGCCGGGC |
| D7BM6/F | CCTGCACCTCTGCAATGATGTCAGTATGACTCTCAGGGACATCCCCCCGGCTGTC |
| D7BM6/R | GACAGCCGGGGGGATGTCCCTGAGAGTCATACTGACATCATTGCAGAGGTGCAGG |
| D7BM7/F | TGATGTCCTCGTCTTGGCCATCAGTATGACTCTCACTGTCACGGGGCAGTGGAAG |
| D7BM7/R | CTTCCACTGCCCCGTGACAGTGAGAGTCATACTGATGGCCAAGACGAGGACATCA |
| D7BM8/F | GGGACATCCCCCCGGTCAGTATGACTCTCAGGAAGCTGTCTGACC |
| D7BM8/R | GGTCAGACAGCTTCCTGAGAGTCATACTGACCGGGGGGATGTCCC |
| D7BM9/F | CTGTCACGGGGCAGTTCAGTATGACTCTCATCCGGCGCTACGGGG |
| D7BM9/R | CCCCGTAGCGCCGGATGAGAGTCATACTGAACTGCCCCGTGACAG |
| D7BM10/F | GGAAGCTGTCTGACCTCAGTATGACTCTCACCGTGCCAAGCGGAT |
| D7BM10/R | ATCCGCTTGGCACGGTGAGAGTCATACTGAGGTCAGACAGCTTCC |
| D7BM11/F | TCCGGCGCTACGGGGTCAGTATGACTCTCATCATCTTTGAAGGCG |
| D7BM11/R | CGCCTTCAAAGATGATGAGAGTCATACTGACCCCGTAGCGCCGGA |
| D7BM12/F | CCGTGCCAAGCGGATTCATCAGTATGACTCTCAGTATGTGTGGGTACTGTAAGTA |
| D7BM12/R | TACTTACAGTACCCACACATACTGAGAGTCATACTGATGAATCCGCTTGGCACGG |
| **Site-directed mutagenesis of pcDNA-human-*DOK7* to make Mut-1 to -7 (QuikChange method)** | |
| Mut-1/F | GGACCAGGTCGAAGTACTGTAAGTA |
| Mut-1/R | TACTTACAGTACTTCGACCTGGTCC |
| Mut-2/F | GGACCAGGTGTGGGTACTGTGGATACGGATGTGTGGGGTCACT |
| Mut-2/R | AGTGACCCCACACATCCGTATCCACAGTACCCACACCTGGTCC |
| Mut-3/F | GGCCGTGCCAAGCGGATAGAATTTTGAAGGCGGGACC |
| Mut-3/R | GGTCCCGCCTTCAAAATTCTATCCGCTTGGCACGGCC |
| Mut-4/F | CCAAGCGGATTCATCTCAGTATGCGGGACCAGGTGTG |
| Mut-4/R | CACACCTGGTCCCGCATACTGAGATGAATCCGCTTGG |
| Mut-5/F | GATTCATCTTTGAAGCCAGAATCAGGTGTGGGTACTG |
| Mut-5/R | CAGTACCCACACCTGATTCTGGCTTCAAAGATGAATC |
| Mut-6/F | CTTTGAAGGCGGGACACTGTAAGTGTACTGTAAGTACGG |

| | |
|---|---|
| Mut-6/R | CCGTACTTACAGTACACTTACAGTGTCCCGCCTTCAAAG |
| Mut-7/F | TTCATCTTTGAAGCCAGAATACTGTAAGTGT |
| Mut-7/R | ACACTTACAGTATTCTGGCTTCAAAGATGAA |
| **Construction of pcDNA-human-*DOK7*-MS2 for tethering analysis (QuikChange method)** | |
| *DOK7* MS2 MG/F | CGGGGCCGTGCCAAGCGGATACATGAGGATCACCCATGTCAGGTGTGGGT ACTGTAAGT |
| *DOK7* MS2 MG/R | ACTTACAGTACCCACACCTGACATGGGTGATCCTCATGTATCCGCTTGGCA CGGCCCCG |
| **Construction of pEGFP-*DOK7*-T-var1** | |
| T-Var1/F | AGTTAAAGCTTCACCATGACCGAGGCGGCGCTG (HindIII) |
| T-Var1/R | CATGTGGTACCGTAGGAGGGGGGTTTAC (KpnI) |
| **Construction of pEGFP-*DOK7*-T-var2** | |
| T-Var2/F | AGTTAAAGCTTCACCATGACCGAGGCGGCGCTG (HindIII) |
| T-Var2/R | CATGTGGTACCGTTCCCCTCCACTGCCC (KpnI) |
| **Deletion of 11 nt sequence from pEGFP-*DOK7*-T-var2 (QuikChange method)** | |
| T-Var2_11/F | TGAAGGCGGGACCAGGGGCTGGCGTCTTC |
| T-Var2_11/R | GAAGACGCCAGCCCCTGGTCCCGCCTTCA |

Restriction sites are underlined and the cognate restriction enzymes are shown in parentheses.

**Supplementary Table S2. Primer sequences for splicing analyses**

| Primers | 5'– 3' sequences |
|---|---|
| Splicing analysis of endogenous *DOK7* | |
| D7 splicing/F | GTGCCAAGCGGATTCATCTT |
| D7 splicing/R | ACAGGAAGCTGATCTGCTCC |
| Splicing analysis of pcDNA-human-*DOK7* | |
| D7 splicing/F | GTGCCAAGCGGATTCATCTT |
| BGH/R | TAGAAGGCACAGTCGAGG |
| Splicing analysis of Blocks 11 and 12 | |
| Splicing 11_12/F | CTGACCTCCGGCGCTACG |
| Splicing 11_12/R | CTCGGCCGAGGACAGGAA |

**Supplementary Table S3. Primer sequences for real-time RT-PCR analysis**

| **Primers** | **5'– 3' sequences** |
|---|---|
| *SRSF1*/F | CCAGACATCCGAACCAAGGA |
| *SRSF1*/R | GCAGACGGTACCCATCGTAA |
| *GAPDH*/F | TGATGACATCAAGAAGGTGGTGAAG |
| *GAPDH*/R | TCCTTGGAGGCCATGTGGGCCAT |

**Supplementary Table S4. Oligonucleotide sequences for synthesizing biotinylated RNA probes**

| Oligonucleotides | 5'– 3' sequences |
|---|---|
| Block 12 Wt | |
| *DOK7* Wt RAP/F | TAATACGACTCACTATAGGGATCTTTGAAGGCGGGACCAGGTGTGGG |
| *DOK7* Wt RAP/R | CCCACACCTGGTCCCGCCTTCAAAGATCCCTATAGTGAGTCGTATTA |
| Block 12 Mut-5 | |
| *DOK7* Mut-5 RAP/F | TAATACGACTCACTATAGGGATCTTTGAAGCCAGAATCAGGTGTGGG |
| *DOK7* Mut-5 RAP/R | CCCACACCTGATTCTGGCTTCAAAGATCCCTATAGTGAGTCGTATTA |
| Block 12 ΔMut-5 | |
| *DOK7* ΔMut-5 RAP/F | TAATACGACTCACTATAGGGATCTTTGAAGCAGGTGTGGG |
| *DOK7* ΔMut-5 RAP/R | CCCACACCTGCTTCAAAGATCCCTATAGTGAGTCGTATTA |

T7 promoter sequence is underlined.
